# Auxilin in enteroendocrine cells controls intestinal homeostasis through inter-cell signaling relays

**DOI:** 10.1371/journal.pgen.1012353

**Published:** 2026-10-26

**Authors:** Runqi Wang, Zhengran Li, Jing Wei, Jiajia Wang, Xiyue Liu Tao, Danjie Zhang, Ruiyan Kong, Xuejing Ren, Hang Zhao, Zhouhua Li

**Affiliations:** Laboratory of Stem Cell Biology, College of Life Sciences, Capital Normal University, Beijing, China; Texas A&M University School of Medicine, UNITED STATES OF AMERICA

## Abstract

Adult stem cells are critical for the homeostasis of residential tissue and organism fitness. Residential stem cells must constantly perceive external and internal signals and adjust their proliferation rate accordingly to avoid unlimited proliferation or premature differentiation. However, how differentiated cells affect stem cell proliferation and tissue homeostasis through cell-cell communication still remains poorly understood. Here, we unveil an unprecedented function of Auxilin (Aux) in enteroendocrine cells (EEs) by restricting intestinal stem cell (ISC) proliferation. *aux* depletion in all EEs leads to intestinal homeostasis disruption. The levels of Slit are dramatically reduced in *aux*-depleted EEs and cytokine Upd3 expression is significantly increased in some *aux*-depleted EEs and neighboring wild type enterocytes (ECs). Ectopic expression of *slit* could effectively rescue the defects observed in *pros*^*ts*^ *> aux*^*RNAi*^ intestines. Interestingly, EGFR signaling is ectopically activated due to EGFR accumulation in *aux*-depleted EEs. Further inhibiting EGFR signaling completely suppresses the defects in *pros*^*ts*^ *> aux*^*RNAi*^ intestines. Finally, EGFR signaling suppresses *slit* transcription by Pointed binding to *slit* regulatory regions. Together, these data show that Aux in EEs restrains ISCs from excessive proliferation and aberrant progeny differentiation to maintain intestinal homeostasis through inter-cell communications. Thus our study uncovers the underlying mechanism of how stem cell proliferation is properly controlled by differentiated cells through inter-cell signaling relays during tissue homeostasis and tumorigenesis.

## Author summary

Adult stem cells are responsible for residential tissue homeostasis. Stem cells must constantly adjust their proliferation rate according to extrinsic and intrinsic signals to maintain tissue homeostasis. However, how stem cell proliferation is controlled by differentiated progeny remains largely unknown. We reveal that Auxilin (Aux) in enteroendocrine cells (EEs) restrains intestinal stem cell (ISC) proliferation and progeny differentiation through inter-cell communications under physiological conditions. *aux* depletion in all EEs disrupts intestinal homeostasis. The expression of *slit* in *aux*-depleted EEs is diminished and the expression levels of cytokine Upd3 are significantly in neighboring wild type enterocytes (ECs). Ectopic EGFR signaling suppresses *slit* expression in *aux*-depleted EEs. Excessive ISC proliferation and aberrant progeny proliferation due to compromised Slit-Robo signaling and activated JAK/STAT signaling contribute to intestinal homeostasis disruption in the absence of Aux in all EEs. Our study shed light on the underlying mechanism of how ISC proliferation and progeny differentiation are properly controlled by differentiated progeny through inter-cell communications during tissue homeostasis and pathogenesis.

## Introduction

To maintain residential tissue homeostasis, adult stem cells must constantly perceive external signals and internal conditions and adjust their proliferation rate accordingly to differentiate and replenish the lost cells. Disruption of the balanced stem cell proliferation and progeny differentiation will lead to excessive stem cell accumulation or precocious stem cell depletion [1–4]. Organism fitness is closely affected by the physiological conditions of adult gastrointestinal tract which is frequently replenished with differentiated progeny of intestinal stem cells (ISCs). However, how differentiated cells in turn affect stem cell proliferation and tissue homeostasis through cell-cell communication remains poorly understood. Thus, illuminating the underlying mechanisms controlling inter-cell communication between differentiated cells and residential stem cells is important for tissue homeostasis control and pathogenesis.

The adult *Drosophila* posterior midgut is an ideal model to study stem cell regulation and cell-cell communication. Equivalent to the mammalian small intestine, the *Drosophila* posterior midgut shares similar characters in terms of development, cellular make-up, and genetic control with its mammalian counterparts [5–8]. The *Drosophila* midgut epithelium is a monolayer of epithelial cells mainly composed of absorptive enterocytes (ECs), with intestinal stem cells (ISCs), enteroblasts (EBs), EE progenitors (EEPs), and enteroendocrine cells (EEs) interspersed along the basement membrane [9–11]. Through asymmetric divisions, ISCs self-renew and produce EBs and/or EEPs which are further differentiated into ECs and EEs, respectively [9–14]. ISCs can also divide symmetrically to replenish lost ISCs [15,16]. The proliferation and differentiation of ISCs under normal and stressed conditions are regulated by multiple signaling pathways such as Notch and EGFR (see reviews by [17–21] and references therein). EEs constitute an important intestinal cell type in the *Drosophila* intestine. Unlike the mammalian secretory lineage which is differentiated into Paneth cells, goblet cells, EEs, and tuft cells, a hierarchical transcription factor cascade regulates EE cell diversity and plasticity in *Drosophila* intestine [22,23]. Slit-Robo signaling originated from EEs had been reported to regulate cell fate decisions in the ISC lineage in *Drosophila* [13,14]. Further, enteroendocrine hormones such as Tachykinin (Tk) and Bursicon (Burs) secreted from some specific subtypes of EEs were found to function as local regulators of ISC proliferation and intestinal homeostasis by modulating the expression of insulin-like factor or EGF in the mesenchymal stem cell niche of the *Drosophila* midgut [24–26]. However, it remains largely unknown how EEs communicate with ISCs to control ISC proliferation and progeny differentiation, thereby maintaining intestinal homeostasis.

Auxilin (Aux), a DnaJ-domain-containing chaperone, functions in the disassembly of Clathrin coated vesicles (CCVs) together with the ATPase Hsc70 and other steps of the CCV cycle [27–30]. Two Aux isoforms exist in vertebrates, the brain-specific Aux 1 and the ubiquitous Aux 2 (or GAK, cyclin G-associated kinase), while only one *Drosophila* Aux ortholog is found which is structurally similar to GAK [28,29]. Previous studies found that Aux functions in the signal-sending cells to regulate Notch signaling in the imaginal discs in *Drosophila* [31–33]. It was reported that GAK plays pivotal role in regulating EGFR trafficking and receptor signaling in mammalian cell lines [34]. Our previous study showed that Aux actively clears EGFR from the plasma membrane (PM) of ISCs through endocytosis to restrain EGFR signaling, thereby maintaining intestinal homeostasis under normal conditions [35]. SH3Ps were reported to recruit Aux to endosomes recently [36]. On the other hand, Aux was reported to facilitate membrane traffic in the early secretory pathway and is required for the formation of Golgi-derived CCVs during *Drosophila* spermatogenesis [30,37]. Our recent work showed that Aux in ECs maintains EC integrity by mediating ER-to-GA trafficking of cell adhesion molecules (CAMs), thereby restraining ISCs from excessive proliferation [38]. Aux-depleted ECs secrete cytokine Upd3 to activate JAK/STAT signaling in ISCs to promote excessive ISC proliferation [38]. However, it remains elusive whether and how Aux functions in EE-ISC communication to control ISC proliferation and progeny differentiation. Here, we show that Aux in EEs restricts ISC proliferation and progeny differentiation via at least two signal relays from EEs to ISCs, thereby maintaining intestinal homeostasis. Thus, our data uncover the underlying mechanism of how ISC proliferation is properly controlled by differentiated cells through inter-cell communications during tissue homeostasis and pathogenesis.

## Results

### Aux is required in all EEs for intestinal homeostasis

Our recent studies showed that the J-domain-containing chaperone Auxilin (Aux) functions in ISCs through Clathrin-mediated trafficking of EGFR to restrain ISC proliferation for tissue homeostasis and participates in ER-Golgi transport of cadherin molecules in enterocytes (ECs) to maintain EC integrity for intestinal homeostasis [35,38]. Using Aux-specific antibody [35,38], we found that, in addition to progenitors and ECs, Aux is also expressed in all EEs (S1 Fig). To investigate whether Aux plays any roles in EEs and intestinal homeostasis, we specifically depleted *aux* in EEs using different effective RNAi constructs against *aux* by the pan-EE driver *pros*^*V1*^*Gal4* (*pros*^*ts*^)(S2 Fig) [35,38]. Interestingly, we found that *pros*^*ts*^ *> aux*^*RNAi*^ flies was shorter lived than control animals, indicative of essential role of Aux in EEs (S2A Fig). Upon closer examination of these intestines, we found that depletion of *aux* in EEs resulted in significant increase in the size of the whole intestine and dramatic increase in the number of intestinal cells in comparison to different control intestines, and the possibility that the observed defects are caused by leaky expression of the RNAi lines in a Gal4-independent manner was excluded (Figs 1A and S2B–S2F, S3). Together, these data suggest that Aux may be required in all EEs for intestinal homeostasis and loss of Aux in EEs will lead to pathogenesis.

**Fig 1:**
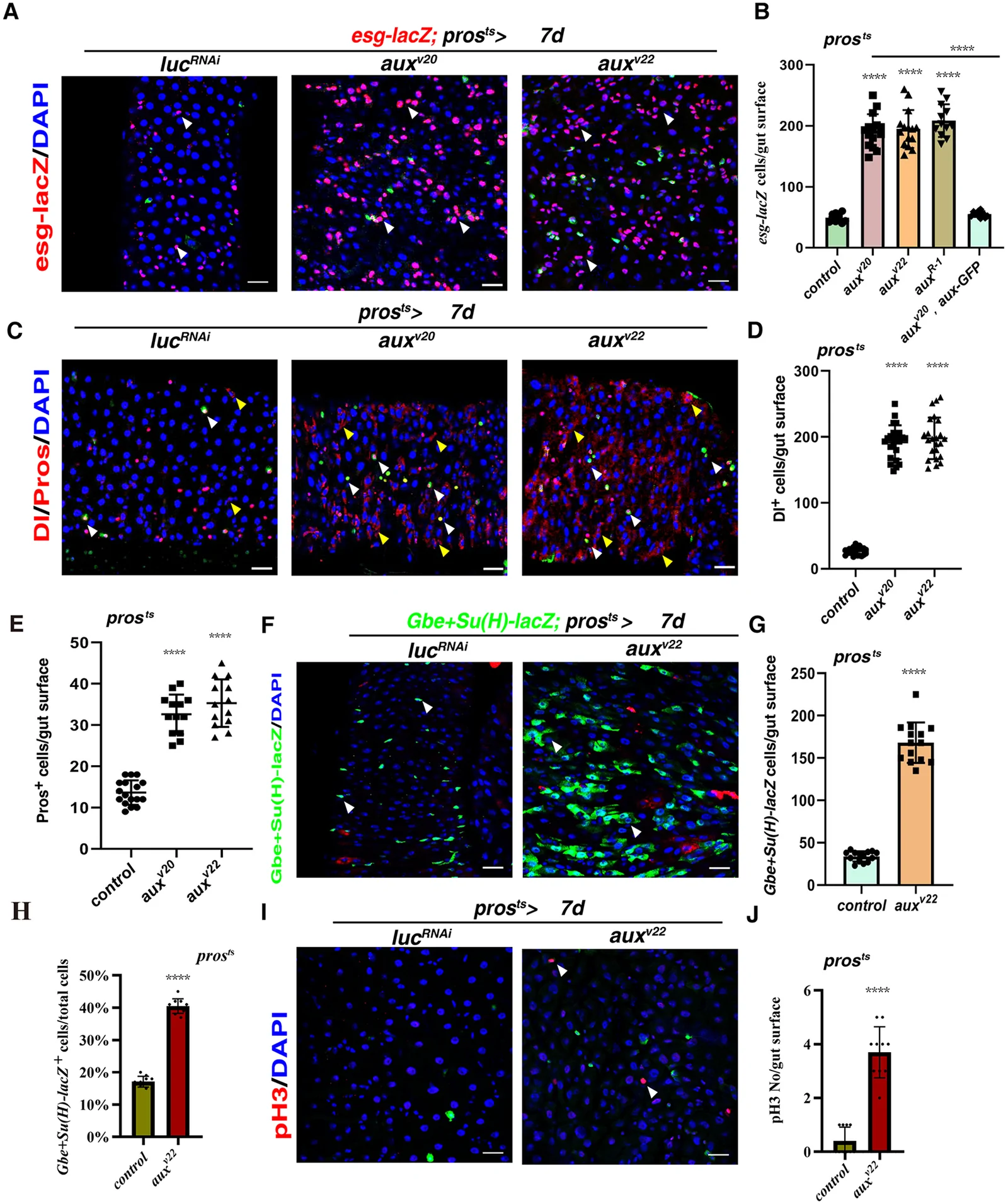
Aux functions in EEs to maintain intestinal homeostasis. (A) Compared to control intestines (*pros*^*ts*^ *> luc*^*RNAi*^), the number of progenitors (by *esg-lacZ* in red, white arrowheads) is dramatically increased in *pros*^*ts*^ *> aux*^*RNAi*^ intestines at 29°C for 7 days. *pros>GFP* cells are in green. The white line indicates the diameter of intestines with indicated genotypes. (B) Quantification of the number of *esg-lacZ*^*+*^ cells/gut surface in different genotypes indicated. Mean ± SD is shown. n ≥ 10 images, ****p < 0.0001. Please note that *pros*^*ts*^ *> aux*^*RNAi*^ intestines are highly deformed preventing accurate quantification of the number of *esg-lacZ*^*+*^ cells. (C) Compared to control intestines (*pros*^*ts*^ *> luc*^*RNAi*^), the number of ISCs (by Dl in red, yellow arrowheads) and EEs (by Pros in red, white arrowheads) is significantly increased in *pros*^*ts*^ *> aux*^*RNAi*^ intestines. (D) Quantification of the number of Dl^+^ cells/gut surface in intestines with indicated genotypes. Mean ± SD is shown. n ≥ 10 images, ****p < 0.0001. Please note that *pros*^*ts*^ *> aux*^*RNAi*^ intestines are highly deformed preventing accurate quantification of the number of Dl^*+*^ cells. (E) Quantification of the number of Pros^+^ cells/gut surface in intestines with indicated genotypes. Mean ± SD is shown. n ≥ 10 images, ****p < 0.0001. (F) Compared to control intestines (*pros*^*ts*^ *> luc*^*RNAi*^), the number of EBs (by *Gbe + Su(H)-lacZ* in green, white arrowheads) is dramatically increased in *pros*^*ts*^ *> aux*^*RNAi*^ intestines at 29°C for 7 days. *pros>RFP* cells are in red. (G) Quantification of the number of *Gbe + Su(H)-lacZ*^*+*^ cells/gut surface in different genotypes indicated. Mean ± SD is shown. n ≥ 10 images, ****p < 0.0001. Please note that *pros*^*ts*^ *> aux*^*RNAi*^ intestines are highly deformed preventing accurate quantification of the number of *Gbe + Su(H)-lacZ*^*+*^ cells. (H) Quantification of the percentage of *Gbe + Su(H)-lacZ*^+^ cells in different genotypes indicated. Mean ± SD is shown. n ≥ 10 images, ****p < 0.0001. (I) Compared to control intestines (*pros*^*ts*^ *> luc*^*RNAi*^), the number of mitotic cells (by pH3 in red, white arrowheads) is significantly increased in *pros*^*ts*^ *> aux*^*RNAi*^ intestines at 29°C for 7 days. *pros>GFP* cells are in green. (**J**) Quantification of the number of pH3^*+*^ cells/gut surface in different genotypes indicated. Mean ± SD is shown. n ≥ 10 images, ****p < 0.0001. The nucleus is labelled by DAPI in blue. Scale bars, 20 μm.

We then determined the identities of the increased cells observed in *pros*^*ts*^ *> aux*^*RNAi*^ intestines. *esg-lacZ* is expressed in progenitors which are infrequently observed in isolation or in clusters containing fewer than three cells in control intestines, however, the number of *esg-lacZ*^*+*^ cells was significantly increased in *pros*^*ts*^ *> aux*^*RNAi*^ intestines, these cells formed large clusters with varied cell sizes and many of them were polyploid, indicative of intestinal homeostasis disruption (Figs 1A, 1B, S3, and S4A). To further confirm that the defects are specific to *aux* depletion, we found that co-expression of an *UAS-aux-flag-GFP* transgene with *aux*^*RNAi*^ construct completely restored midgut homeostasis as the number of *esg-lacZ*^*+*^ cells was restored to normal (Figs 1B and S4A) [35]. Next, we stained the intestines with antibodies against Dl (an ISC marker) and Pros (an EE marker). The number of Dl^+^ cells in *pros*^*ts*^ *> aux*^*RNAi*^ intestines was significantly increased compared to those in control flies (Figs 1C, 1D, and S4B). The great increase in the number and percentage of Dl^+^ cells was independently verified with a *Dl-lacZ* reporter line (S4C Fig). The sizes of these *Dl-lacZ*^+^ cells were heterogeneous in size, indicating that they are ISCs, ISC-like cells, and/or EBs (S4C Fig). Consistent with the significant increase of *pros>GFP*^+^ cells, the number of Pros^+^ cells was also significantly increased in *pros*^*ts*^ *> aux*^*RNAi*^ intestines (Figs 1C, 1E, and S1C). Further, the number and percentage of EBs (by *Gbe+Su(H)-lacZ*) were dramatically increased in *pros*^*ts*^ *> aux*^*RNAi*^ intestines compared to those in control flies (Fig 1F-1H). The number and percentage of ECs were not determined. Finally, significant increase in the number of mitotic cells was observed in *aux-*depleted intestines, suggesting that the dramatic increase of intestinal cells is caused by excessive ISC proliferation and aberrant differentiation (Fig 1I and 1J). Collectively, these data show that Aux in EEs may restrict ISC proliferation and progeny differentiation in a non-cell autonomous manner to maintain intestinal homeostasis under physiological conditions.

Although Pros is weakly detected in some ISCs, previous reports and our study showed that this driver is specifically expressed in all EEs, but not in ISCs [24,39–42]. To further exclude the possibility that the ISC proliferation phenotype observed in *pros*^*ts*^ *> aux*^*RNAi*^ intestines is resulted from consequences of *aux* knockdown in ISCs rather than EEs, we manipulated components from the Notch and JAK/STAT signaling pathway [43,44]. Previous studies showed that depleting Notch in ISCs led to rapid ISC proliferation and progeny differentiation defects, resulting in cell clusters composed of ISCs (or ISC-like cells) and/or EEs with almost identical cell size, while overexpression of constitutively active form of Hop (Hop^TumL^) in ISCs results in rapid ISC proliferation [9,45,46]. However, we did not observe the expected phenotypes as they are manipulated by *esgGal4* in progenitors, on the contrary, the *pros*^*ts*^*>Notch*^*RNAi*^ and *pros*^*ts*^ *> hop*^*TumL*^ intestines were largely normal as those of control flies (S5 Fig). These results show that the observed phenotypes in *pros*^*ts*^ *> aux*^*RNAi*^ intestines are indeed resulted from *aux* depletion in EEs, rather than in ISCs. *prosGal4* driver is also expressed in the central nervous system (CNS) [47]. Theoretically, to exclude the possibility that the defects observed in *pros*^*ts*^ *> aux*^*RNAi*^ intestines are resulted from *aux* depletion in the CNS, rather than EEs, *prosGal4* expression in the CNS could be blocked using tools like nSybGal80. Unfortunately, previous studies showed that *nSybGal4* is also expressed in most EEs in addition to its expression in the CNS [48,49]. Therefore, combination of nSybGal80 with *prosGal4* will inhibit Gal4 activity in both the CNS and most EEs, leading to no *aux* depletion in most EEs, thus preventing any meaningful conclusion drawn. Our results further confirmed that depletion of Aux by *prosGal4* in the presence of nSybGal80 resulted in no obvious defects (S6A Fig). Thus we suppressed brain expression of *prosGal4* by ChATGal80 [26], the results showed that the number of *esg-lacZ*^+^ cells is still significantly increased in these intestines, mimicking those of *pros*^*ts*^ *> aux*^*RNAi*^ intestines (S7 Fig). These data show that the defects observed in *pros*^*ts*^ *> aux*^*RNAi*^ intestines are very unlikely caused by *aux* knockdown in the CNS rather than EEs. To further exclude the possibility the observed defects are resulted from *aux* knockdown in ISCs rather than EEs, we verified the phenotype by using a newly identified pan-EE driver, *CG32547Gal4* [42]. The *CG32547*^*ts*^*>aux*^*RNAi*^ intestines are similar as those of *pros*^*ts*^ *> aux*^*RNAi*^ intestines: increased ISC proliferation and accumulation of intestinal cells (S6B Fig). Altogether, these data show that the observed phenotype of *pros*^*ts*^ *> aux*^*RNAi*^ intestines are likely resulted from *aux* depletion in EEs rather than other cell types.

Moreover, similar as their mammalian counterparts, EEs are also diversified into considerable subtypes in *Drosophila* intestines [23,42], therefore, we asked whether Aux is required in all EEs or certain EE subtype(s) for intestinal homeostasis. We further characterized the percentage of certain EE subtypes over total EEs using subtype-specific EE drivers like *386YGal4*, *amonGal4*, and *tkGal4*. We found that these subtype-specific EEs account for less than 50% of all EEs in the R5 region of intestine where we examined (S8 Fig). Unsurprisingly, no obvious defects were observed when *aux* was depleted in certain EE subtypes using drivers such as *tkGal4* or *386YGal4*, further supporting the notion that Aux functions in all EEs to maintain intestinal homeostasis rather than in certain EE subtypes (S9 Fig). Collectively, these data show that Aux functions in all EEs to maintain intestinal homeostasis under physiological conditions.

### Defective Slit-Robo signaling and activated JAK/STAT signaling are responsible for the defects observed upon Aux depletion

Next, we investigated how intestinal homeostasis was disrupted in *pros*^*ts*^ *> aux*^*RNAi*^ intestines. Previous studies reported that EEs secrete Slit to activate Slit-Robo signaling in ISCs to restrain EE differentiation and ISC proliferation [13,14,24,25,41], the increase of EEs in *pros*^*ts*^ *> aux*^*RNAi*^ intestines promoted us to examine whether Slit-Robo signaling is affected upon *aux* depletion in EEs. Slit is synthesized and secreted from EEs and binds to Robo2 receptor in progenitors (Fig 2A) [13,14]. However, the protein levels of Slit in *aux*-depleted EEs were diminished, indicating that Slit production in EEs is affected (Fig 2A). Consistent with the perception of Slit by progenitors, Slit proteins could be barely detectable in progenitors of *pros*^*ts*^ *> aux*^*RNAi*^ intestines (Fig 2A). These data suggest that Aux may function through Slit-Robo signaling to maintain tissue homeostasis. Although previous studies reported that Slit-Robo signaling mainly restrains progeny differentiation [13,14,24,25,41], closer examination of previous published results indicated that depleting *slit* in all EEs resulted in accumulation of intestinal cells, indicative of increased ISC proliferation [40]. Consistently, we found that depletion of *slit* in all EEs by *pros*^*ts*^ indeed caused similar, albeit weaker, defects as those in *pros*^*ts*^ *> aux*^*RNAi*^ intestines (S10A Fig). Further, depleting *robo2* in progenitors by *esg*^*ts*^ driver also resulted in accumulation of progenitors and increased ISC proliferation, albeit weaker as those of *pros*^*ts*^ *> aux*^*RNAi*^ intestines (S10B-S10E Figs).

**Fig 2:**
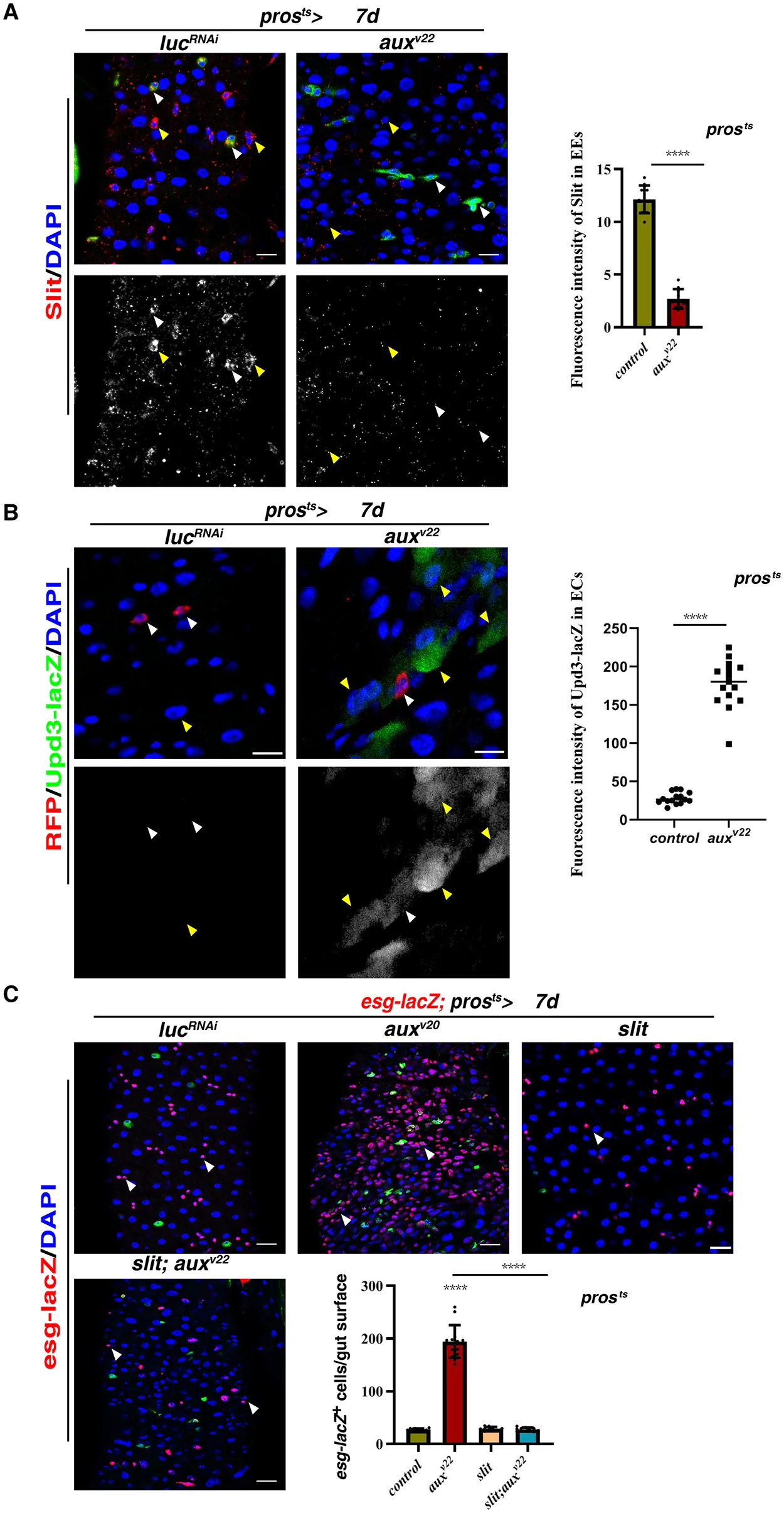
Slit levels are diminished but *Upd3-lacZ* levels are significantly increased in *pros*^*ts*^ *> aux*^*RNAi*^ intestines. (A) Compared to control intestines (*pros*^*ts*^ *> luc*^*RNAi*^), the protein levels of Slit (by Slit in red, white arrowheads) are dramatically decreased in *pros*^*ts*^ *> aux*^*RNAi*^ intestines at 29°C for 7 days. *pros>GFP* cells are in green. Please note that the protein levels of Slit are also diminished in progenitors (yellow arrowheads) of *pros*^*ts*^ *> aux*^*RNAi*^ intestines. Slit channel is showed separately in black white. Quantification of the fluorescence intensity of Slit in EEs in control and *pros*^*ts*^ *> aux*^*RNAi*^ intestines. Mean ± SD is shown. n ≥ 10 cells from different intestines, ****p < 0.0001. (B) Compared to control intestines (*pros*^*ts*^ *> luc*^*RNAi*^) in which *Upd3-lacZ* (green, white and yellow arrowheads) is barely detectable, the levels of *Upd3-lacZ* in neighboring wild type ECs are significantly increased in *pros*^*ts*^ *> aux*^*RNAi*^ intestines at 29°C for 7 days. *pros>RFP* cells are in red (yellow arrowheads). Please note that the levels of *Upd3-lacZ* are also increased in some EEs (white arrowhead) of *pros*^*ts*^ *> aux*^*RNAi*^ intestines. *Upd3-lacZ* channel is showed separately in black white. Quantification of the fluorescence intensity of *Upd3-lacZ* in ECs in control and *pros*^*ts*^ *> aux*^*RNAi*^ intestines. N ≥ 10 cells from different intestines. Mean ± SD is shown. ****p < 0.0001. (C) Ectopic expression of *slit* in *pros*^*ts*^ *> aux*^*RNAi*^ intestines could completely rescue the defects observed in *pros*^*ts*^ *> aux*^*RNAi*^ intestines. The progenitors are indicated by *esg-lacZ* in red (white arrowheads). Quantification of the number of *esg-lacZ*^*+*^ cells/gut surface in different genotypes indicated. Mean ± SD is shown. n ≥ 10 images, ****p < 0.0001. The nucleus is labelled by DAPI in blue. Scale bars, 20 μm (A, C), 10 μm (B).

As defective Slit-Robo signaling could not fully recapitulate the defects observed in *pros*^*ts*^ *> aux*^*RNAi*^ intestines, we speculated that some other effector(s) may also contribute to the excessive ISC proliferation and aberrant progeny differentiation upon Aux depletion in all EEs. Interestingly, we found that the expression of *Upd3-lacZ*, one of the important ligand of JAK/STAT signaling, is significantly upregulated in some *aux*-depleted EEs and neighboring wild type ECs (Fig 2B). As previous publications and our study already showed that paracrine Upd3 from dying ECs could drive excessive progenitor proliferation by activating JAK/STAT signaling in ISCs under stress conditions and *aux* depletion in ECs [38,46,50], these data indicate that the great accumulation of progenitors and intestinal homeostasis disruption are likely due to synergetic effects of defective Slit-Robo signaling and activated JAK/STAT signaling. To examine this hypothesis, we performed rescue experiments. Interestingly, simultaneous expression of *slit* in *pros*^*ts*^ *> aux*^*RNAi*^ intestines effectively restored the defects observed in *pros*^*ts*^ *> aux*^*RNAi*^ intestines determined by *esg-lacZ* (Fig 2C). The effective rescue capability of Slit in *pros*^*ts*^ *> aux*^*RNAi*^ intestines also indicate that Aux plays minor or no roles in the exocytosis pathway as it is required in ECs for the secretion of cell adhesion molecules to maintain EC integrity (S11 Fig) [38]. Altogether, these data indicate that defective Slit-Robo signaling and activated JAK/STAT signaling are responsible for increased ISC proliferation and aberrant progeny differentiation upon Aux depletion in all EEs.

### Aux quenches EGFR signaling by mediating EGFR internalization in EEs

We then determined the underlying mechanisms by which Aux non-cell autonomously restrains ISC proliferation and progeny differentiation. Previous studies showed that Aux mediates internalization of various receptors like Dl and EGFR on the plasma membrane (PM) [31–33,35], we investigated whether Aux is also involved in endocytosis of certain receptor(s) on the PM of EEs. We examined the activity of EGFR signaling in intestines. EGFR signaling is activated in progenitors, but not in other cell types in control intestines (Fig 3A). Unexpectedly, we found that EGFR signaling was ectopically activated in *aux*-depleted EEs, indicating that Aux is required to quench EGFR signaling in EEs under physiological conditions (Fig 3A and 3B). As EGFR is activated by binding to EGFs on the PM, we asked whether Aux affects EGFR internalization using an EGFR-specific antibody [35]. In control intestines, EGFR is mainly localized in cytosolic puncta in progenitors, while EGFR signal was undetectable in EEs (Fig 3C). In contrast, EGFR proteins were unexpectedly appeared on/near the PM of EEs in *pros*^*ts*^ *> aux*^*RNAi*^ intestines (Fig 3C and 3D). The unexpected appearance of EGFR protein in *aux*-depleted EEs was further verified by an *EGFR-GFP* reporter line (S12 Fig). These data indicate that Aux may facilitate the internalization of EGFR through endocytosis in EEs under normal situations. Consistent with this notion, the number of progenitors was also significantly increased and intestinal homeostasis was disrupted when endocytosis was blocked in EEs (S13A Fig). Consistently, EGFR proteins were also ectopically enriched in *Avl*-depleted EEs, similar as to those of *aux*-depleted EEs (Figs 3C and S13B). Together with our previous study that Aux associates with and mediates the internalization of EGFR in progenitors [35], these data show that Aux promotes EGFR endocytosis to quench EGFR signaling in EEs under normal situations.

**Fig 3:**
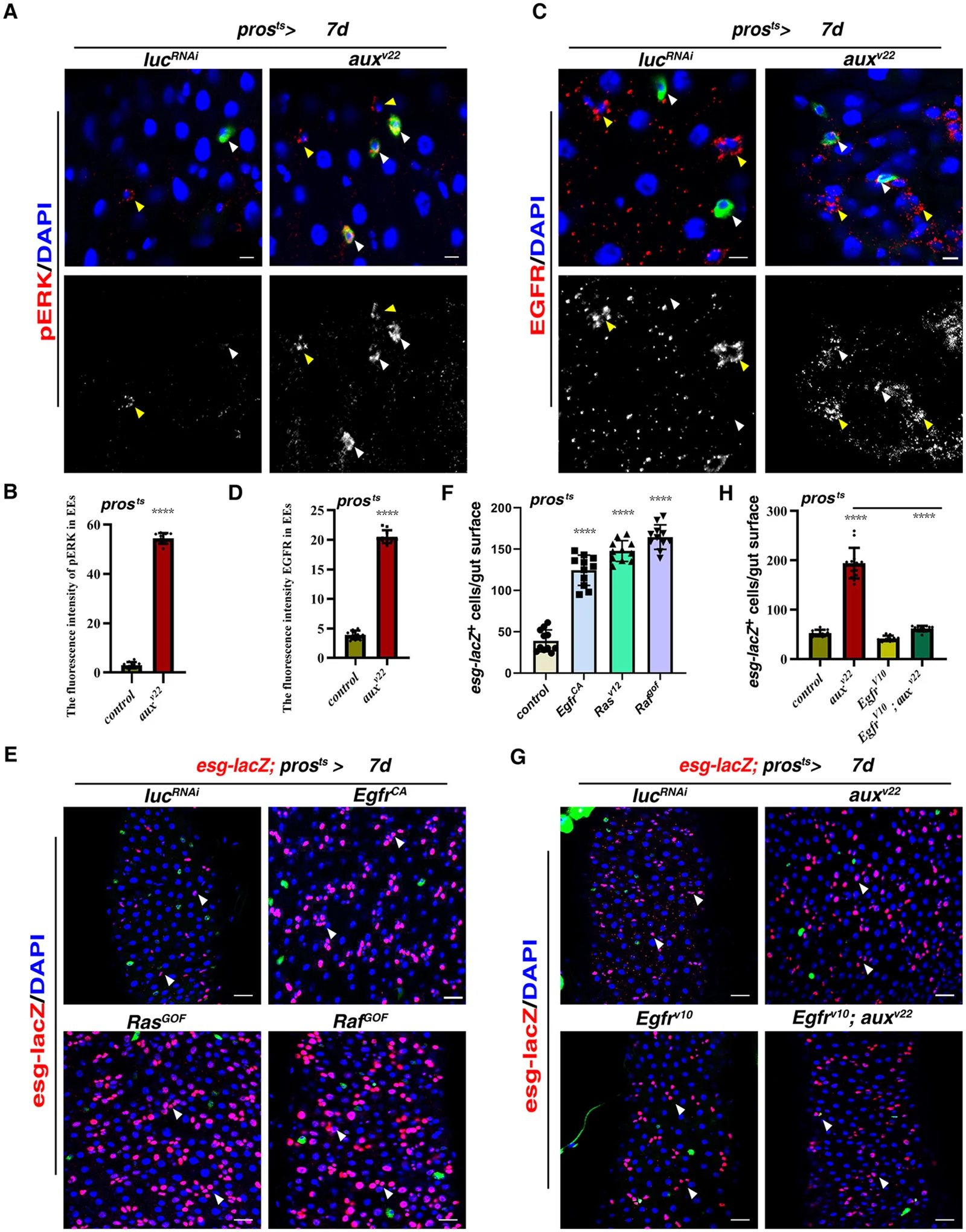
Ectopically activated EGFR signaling is responsible for the defects observed in *pros*^*ts*^ *> aux*^*RNAi*^ intestines. (A) Compared to control intestines in which EGFR signaling (by pERK in red) is barely detectable in EEs, EGFR signaling is dramatically activated in EEs of *pros*^*ts*^ *> aux*^*RNAi*^ flies (white arrowheads) at 29°C for 7 days. pERK signal in progenitors/ISCs is indicated by yellow arrowheads. pERK channel is showed separately in black white. (B) Quantification of the fluorescence intensity of pERK in EEs in control and *pros*^*ts*^ *> aux*^*RNAi*^ intestines. Mean ± SD is shown. n ≥ 10 cells from different intestines, ****p < 0.0001. (C) Compared to control intestines in which EGFR (by EGFR in red) is barely detectable in EEs, the protein levels of EGFR are significantly increased in EEs of *pros*^*ts*^ *> aux*^*RNAi*^ flies (white arrowheads) at 29°C for 7 days. EGFR in progenitors/ISCs is indicated by yellow arrowheads. EGFR channel is showed separately in black white. (D) Quantification of the fluorescence intensity of EGFR in EEs in control and *pros*^*ts*^ *> aux*^*RNAi*^ intestines. Mean ± SD is shown. n ≥ 10 cells from different intestines, ****p < 0.0001. (E) Ectopic activation of EGFR signaling in EEs leads to great increase of progenitors (by *esg-lacZ* in red, white arrowheads) at 29°C for 7 days. (F) Quantification of the number of *esg-lacZ*^*+*^ cells/gut surface in different genotypes indicated. Mean ± SD is shown. n ≥ 10 images, ****p < 0.0001. (G) Simultaneous depletion of *Egfr* in *pros*^*ts*^ *> aux*^*RNAi*^ intestines could completely rescue the dramatic increase of progenitors (by *esg-lacZ* in red, white arrowheads) observed in *pros*^*ts*^ *> aux*^*RNAi*^ intestines. (H) Quantification of the number of *esg-lacZ*^*+*^ cells/gut surface in different genotypes indicated. Mean ± SD is shown. n ≥ 10 images, ****p < 0.0001. The nucleus is labelled by DAPI in blue. Scale bars, 20 μm (except A and C, 5 μm).

Next, we investigated whether ectopic EGFR activation in EEs could recapitulate the defects observed in *pros*^*ts*^ *> aux*^*RNAi*^ intestines. Indeed, when EGFR signaling was ectopically activated in all EEs by either means, the number of *esg-lacZ*^+^ cells was significantly increased, these cells formed large clusters with varied cell sizes and many of them were polyploid, reminiscent of *pros*^*ts*^ *> aux*^*RNAi*^ intestines (Fig 3E and 3F). These data suggest that ectopically activated EGFR signaling in *aux*-depleted EEs is likely responsible for the defects observed in *pros*^*ts*^ *> aux*^*RNAi*^ intestines. We further confirmed this conclusion by performing suppression experiments. The results showed that further depletion of EGFR in *aux*-depleted EEs could completely suppress the defects observed in *pros*^*ts*^ *> aux*^*RNAi*^ intestines determined by *esg-lacZ*, indicating that ectopically activated EGFR signaling is mainly responsible for the defects observed in *pros*^*ts*^ *> aux*^*RNAi*^ intestines (Fig 3G and 3H). Altogether, these data demonstrate that Aux promotes EGFR internalization and degradation in EEs to quench EGFR signaling, thus maintaining intestinal homeostasis under physiological conditions.

### EGFR signaling directly suppresses the expression of *slit*

As the protein levels of Slit are diminished in *pros*^*ts*^ *> aux*^*RNAi*^ intestines and further blocking EGFR signaling in *aux*-depleted EEs could completely rescue the defects observed in *pros*^*ts*^ *> aux*^*RNAi*^ intestines, we then examined whether the protein levels of Slit were affected when EGFR signaling was ectopically activated in EEs. Consistent with the notion that ectopically activated EGFR signaling is mainly responsible for the defects observed in *pros*^*ts*^ *> aux*^*RNAi*^ intestines, we found that the protein levels of Slit were also diminished when EGFR signaling was ectopically activated in EEs (Fig 4A and 4B). On the contrary, the levels of Slit protein were significantly increased when EGFR signaling was blocked in EEs (Fig 4A and 4B). Consistently, the protein levels of Slit in progenitors were also significantly increased in *pros*^*ts*^ *> Raf*^*RNAi*^ intestines (Fig 4A). Similarly, the levels of Slit protein were also diminished when *aux* was depleted in EEs or EGFR signaling was ectopically activated in EEs by *CG32547Gal4*, while Slit protein levels were significantly increased in *CG32547Gal4*^*ts*^*>Raf*^*RNAi*^ intestines (S14 Fig). Together, these data indicate that EGFR signaling negatively regulates the production of Slit proteins in EEs.

**Fig 4:**
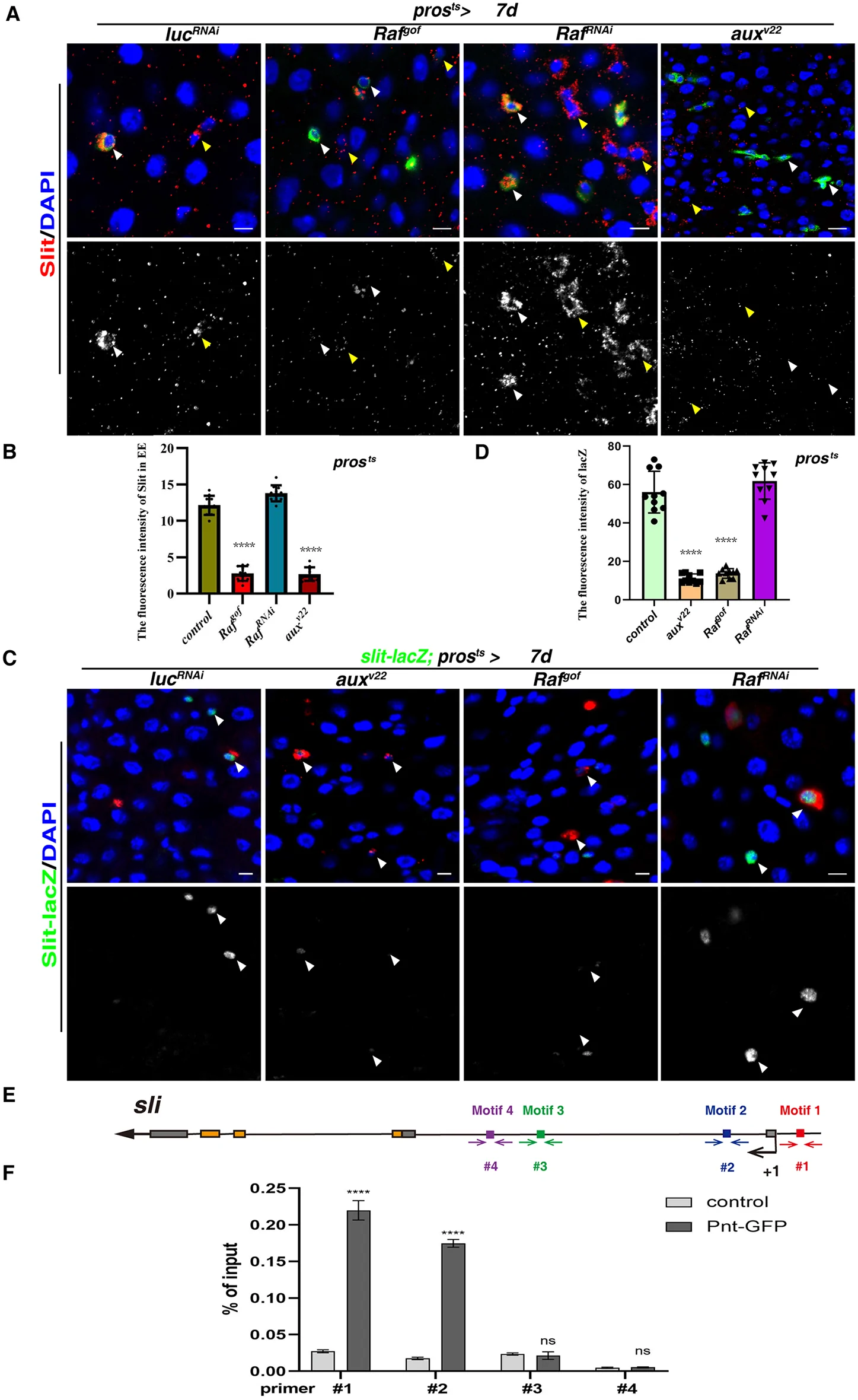
The expression of *slit* is suppressed by EGFR signaling. (A) The protein levels of Slit in EEs (red, white arrowheads) are diminished upon ectopic activation of EGFR signaling in EEs. Slit in progenitors/ISCs is indicated by yellow arrowheads. Slit channel is showed separately in black white. (B) Quantification of the fluorescence intensity of Slit in EEs of intestines with indicated genotype. Mean ± SD is shown. n ≥ 10 cells from different intestines, ****p < 0.0001. (C) Compared to the control, the expression of *slit* (by *slit-lacZ* in green, white arrowheads) in EEs is significantly suppressed in *pros*^*ts*^ *> aux*^*v22*^ and *pros*^*ts*^ *> Raf*^*gof*^ intestines. Whilst the expression of *slit* in EEs is increased in *pros*^*ts*^ *> Raf*^*RNAi*^ intestines. *Slit-lacZ* channel is showed separately in black white. (D) Quantification of the fluorescence intensity of *slit-lacZ* in EEs of intestines with indicated genotype. Mean ± SD is shown. n ≥ 10 cells from different intestines, ****p < 0.0001. (E) Scheme of putative Pointed (Pnt) binding motifs (Motif 1–4 in different colors) in *slit* genomic region with the transcription start site (TSS, + 1) indicated. The primer pairs to detect the putative Pnt binding motifs are indicated. (F) The ChIP-qPCR results of Pnt-GFP show that Pnt binds to Motif 1 and 2 near the TSS of *slit* when *aux* is depleted. *tubulinGal4 > GFP* flies were used as negative control. Mean ± SD is shown. n = 6 replicates, ****p < 0.0001. The nucleus is labelled by DAPI in blue. Scale bars, 5 μm.

We further determined how the Aux-EGFR axis affects the production of Slit proteins in EEs. We first examined whether the transcription of *slit* was affected by the Aux-EGFR axis by a *slit-lacZ* reported line. In control intestines, *slit-lacZ* is only expressed in EEs in which EGFR signaling is undetectable, while the expression of *slit-lacZ* is barely detectable in progenitors in which EGFR signaling is activated, indicating that *slit* transcription may be suppressed by EGFR signaling (Fig 4C) [13,14]. In clear contrast to those in control intestines, the expression of *slit-lacZ* is dramatically reduced in *aux*-depleted EEs, indicating that Aux maintains *slit* expression under normal conditions (Fig 4C and 4D). Consistently, the levels of *slit* transcripts were also significantly reduced in *pros*^*ts*^ *> aux*^*RNAi*^ intestines as determined by RT-qPCR (S15A Fig). However, the expression levels of *slit* were unaffected in the brains of *pros*^*ts*^ *> aux*^*RNAi*^ animals, further excluding the possibility that the defects observed in *pros*^*ts*^ *> aux*^*RNAi*^ intestines are due to *aux* depletion in the CNS rather than in EEs (S15B Fig). Further, the expression of *slit-lacZ* was also dramatically decreased when EGFR signaling was ectopically activated in EEs, phenocopying those of *pros*^*ts*^ *> aux*^*RNAi*^ intestines, suggesting that EGFR signaling suppresses *slit* expression under physiological conditions (Fig 4C and 4D). To further confirm this conclusion, we blocked EGFR signaling in EEs. Supporting this notion, the expression of *slit* is significantly increased in EEs of *pros*^*ts*^ *> Raf*^*RNAi*^ intestines (Fig 4C and 4D).

We further examined the epistasis between EGFR signaling and *slit*. We found the significantly increased number of progenitors observed in *pros*^*ts*^ *> Raf*^*gof*^ intestines was completely suppressed by co-expression of *UAS-slit*, supporting the notion that *slit* acts downstream of EGFR signaling to regulate ISC proliferation and progeny differentiation (S16 Fig). We then determined whether the suppression of *slit* transcription by EGFR signaling could be more broadly applied, we examined the expression of *slit* in progenitors in which *slit-lacZ* is not detectable under normal conditions. Consistent with this conclusion, *slit-lacZ* was ectopically expressed in progenitors when EGFR signaling was blocked in progenitors (S17 Fig). Taken together, these data show that the Aux-EGFR axis regulates the transcription of *slit* to control ISC proliferation and intestinal homeostasis through cell-cell communication.

Finally, we investigated whether EGFR signaling directly suppressed the transcription of *slit* by direct binding of Pointed (Pnt/Ets), the downstream transcription factor of EGFR signaling, to the regulatory regions of *slit* locus. Consistent with the notion that ectopic activation of EGFR signaling in all EEs results in great accumulation of progenitors and intestinal homeostasis disruption, ectopic expression of *pntP1* led to great accumulation of progenitors (S18 Fig). According to previous reports, the conserved Pnt binding motif is **C**/G/T/A **C**/A/G/T **GGA T**/A **G**/A/C **C**/T/A ([51] and references therein). We identified four putative Pnt binding motifs (Motif 1–4) in the regulatory regions of *slit* (from 2 kb upstream of transcription start site/TSS to the huge intron spanning the 5’UTR) (Fig 4E). We then performed chromatin immunoprecipitation (ChIP) of Pnt-GFP followed by quantitative PCR (ChIP-qPCR) in *aux*-depleted animals, with flies expressing GFP as negative control. The ChIP-qPCR results showed that Pnt effectively binds to two of these putative binding motifs (Motif 1 and 2) surrounding the TSS of *slit*, indicating that Pnt directly binds to the regulatory region of *slit* and suppresses the transcription of *slit* when EGFR signaling is activated (Fig 4F). Altogether, these data reveal that EGFR signaling suppresses *slit* expression to control ISC proliferation and progeny differentiation.

## Discussion

In conclusion, we show that under physiological conditions, Aux in EEs associates with EGFR and promotes its internalization and degradation, ensuring traceable or no activation of EGFR signaling in EEs. Thus *slit* can thus be transcribed and Slit proteins are secreted from EEs to activate Robo signaling in ISCs to restrain ISC proliferation and progeny differentiation, thereby maintaining intestinal homeostasis. Whilst in intestines with *aux*-depleted EEs, EGFR can not be internalized and be accumulated on the plasma membrane of EEs, resulting in ectopic EGFR signaling. Upon ectopic activation of EGFR signaling, the transcription factor Pointed binds to the binding motifs near the TSS of *slit* and suppresses the expression of *slit*. Meanwhile, the expression of cytokine Upd3 is significantly increased in some *aux*-depleted EEs and neighboring wild type ECs, which will activate JAK/STAT signaling in ISCs for excessive ISC proliferation. Further, activated EGFR signaling also regulates multiple downstream targets in *aux*-defective EEs, including the yet-to-be identified diffusible factor(s) (termed X). The yet-to-be identified factor X from *aux*-depleted EEs may send signal to ECs, resulting death of ECs which secrete cytokine Upd3 to further activate JAK/STAT signaling in ISCs to promote ISC hyper-proliferation. With the synergetic effects of Upd3-JAK/STAT signaling, the yet-to-be identified diffusible factor X and defective Slit-Robo signaling, ISCs are released from proliferation restraint and undergo excessive proliferation and aberrant progeny differentiation, leading to intestinal homeostasis disruption and pathogenesis (S19 Fig). Therefore, the inter-cell communication cascades downstream of Aux-EGFR by defective Slit-Robo and activated JAK/STAT signaling are likely implicated in tissue homeostasis and tumorigenesis. Consistently, components in the Slit-Robo and JAK/STAT pathway have been suggested to directly participate in tumorigenesis [38,46,52–55]. Based on the conservation of these signaling molecules and mechanisms controlling stem cell proliferation and progeny differentiation, we can anticipate that the inter-cell signal relays may also control tissue homeostasis and pathogenesis in mammals.

Previous studies reported that specific EE subtypes could secrete enteroendocrine hormones such as Tachykinin (Tk) or Bursicon (Burs) to regulate the expression of insulin-like peptide (Ilp) or EGF in the visceral muscles (VMs) respectively, forming a signaling relay process which in turn regulates ISC proliferation and intestinal homeostasis [24–26]. However, depleting *aux* in some subtypes of EEs did not cause obvious defects, indicating that Aux may be more broadly required in EEs. Although we show that the defective Slit-Robo and activated JAK/STAT signaling are mainly responsible for the defects observed in intestines with *aux*-depleted EEs, we could not fully exclude the possibility that additional diffusible signaling molecule(s) from *aux-*defective-EEs may also form a signaling relay cascade like those of Tk/Burs-EGFs/Ilps as reported before and is involved in the regulation of ISC proliferation, intestinal homeostasis and pathogenesis when *aux* is depleted in all EEs [24–26]. Further, the effective rescue of the defects observed in *pros*^*ts*^ *> aux*^*RNAi*^ intestines by ectopic *slit* expression indicates that ectopic Slit-Robo signaling is dominant to the yet-to-be identified factor X and/or can dampen the subsequent JAK/STAT signaling from ECs to ISCs. It will be interesting to dissect the full functions of EEs in intestinal homeostasis, physiology, and pathogenesis in future studies.

## Methods

### Fly lines and cultures

Flies were maintained on standard media at 25 °C. Crosses were raised at 18°C in humidity controlled incubators, or as otherwise noted. Flies hatched in 18°C incubators (2–3 days old) were picked and transferred to 29 °C incubator, unless otherwise specified. Flies were transferred to new vials with fresh food every day, and dissected at time points specified in the text. In all experiments, only the female posterior midgut (the R5 region) was analyzed. Information for alleles and transgenes used in this study can be found either in FlyBase, TRiP stock center at Tsinghua University (THU), or as noted. *pros*^*V1*^*Gal4, UAS-CD8-GFP, tubGal80*^*ts*^ (*pros*^*ts*^), *pros*^*V1*^*Gal4, UAS-RFP, tubGal80*^*ts*^ (*pros*^*ts*^ *> RFP*), *esgGal4, UAS-CD8-GFP, tubGal80*^*ts*^ (*esg*^*ts*^)*, aux*^*v20*^ (*aux*^*RNAi-1*^*,* THU3942/BL39017/HMS01935, constructed on a pVALIUM20 vector), *aux*^*v22*^ (*aux*^*RNAi-2*^*,* THU0189/BL35310/GL00213, constructed on a pVALIUM22 vector), *aux*^*R-1*^ (*aux*^*RNAi-3*^*,* NIG, 1107R-1), *aux*^*R-2*^ (*aux*^*RNAi-4*^*,* NIG, 1107R-2), *aux*^*v10*^ (*aux*^*RNAi-5*^*,* BL 28509/JF03129, constructed on a pVALIUM10 vector) [35,38], *tubGal80*^*ts*^*, tubGal4* (*tub*^*ts*^), *esg-lacZ*^*B7–2-22*^, *tkGal4* (BL39282), *amonGal4* (BL*30554*), *386YGal4* (BL25410), *CG32547Gal4* [42](gift from Dr Rongwen Xi, National Institute of Biological Sciences (NIBS). It is also expressed in the brain and late stage follicles in the ovary.), *Gbe+Su(H)-lacZ* (gift from Dr Sarah Bray, University of Cambridge) [56], *Dl-lacZ* (*Dl*^*05151*^), *UAS-aux-Flag-GFP* [35], *slit-lacZ* (BL12189), *EGFR-GFP* (BL61765), *Egfr*^*RNAi*^ (*Egfr*^*v10*^, THU1939/BL25781, constructed on a pVALIUM10 vector), *Ras85D*^*RNAi*^ (VDRC, v28129/GD12253) [57], *UAS-slit* (BL6472, BL67688), *Raf*^*RNAi*^ (BL41589/GL01125, TH02413.N/BL55679), *slit*^*RNAi*^ (BL31468/JF1229), *robo2*^*RNAi*^ (THU3624/HMS01063, VDRC, GD11823), *Raf*^*gof*^ (BL2033) [58], *Egfr*^*CA*^ (BL9533), *Ras*^*gof*^ (BL4847), *robo2*^*RNAi*^ (THU3624/HMS01063, VDRC, GD11823), *Notch*^*RNAi*^ (NIG, 3936R-2), *UAS-hop*^*TumL*^ [43,59–61], *pnt-GFP* (BL42680. Genomic fragment including the *pnt* gene from BAC clone CH321-39L02 was constructed by Rebecca Spokony and Kevin White, University of Chicago. Tags including GFP and FLAG had been introduced at the C-terminal end of the *pnt* gene. Gift from Dr Yufeng Yang, Fuzhou University) [62], *Dsor1*^*RNAi*^ (THU1111/BL32920), *nSybGal80*, *rl*^*RNAi*^ (THU3530/BL34855/HMS00173), *ChATGal80* (gift from Dr Yan Li, Institute of Biophysics, Chinese Academy of Sciences), *UAS-PntP1* (BL869, gift from Dr Zongzhao Zhai, Hunan Normal University), *luc*^*RNAi*^ (HMS504, from TRiP at Harvard Medical School) and/or *w(white)*^*RNAi*^ (BL33623) were used as control*.*

### RNAi knockdown and overexpression experiments

To address gene function in enteroendocrine cells, *prosGal4, UAS-CD8-GFP, tubGal80*^*ts*^ (*pros*^*ts*^), and/or *pros*^*V1*^*Gal4, UAS-RFP, tubGal80*^*ts*^ (*pros*^*ts*^ *> RFP*) were used, for RNAi knock down efficiency and ChIP-qPCR, *tubGal80*^*ts*^*, tubGal4* (*tub*^*ts*^) was used. To block EGFR signaling in progenitors (by *Raf*^*RNAi*^), *esgGal4, UAS-CD8-GFP, tubGal80*^*ts*^ (*esg*^*ts*^) was used. The crosses (unless stated otherwise) were maintained at 18°C to bypass potential requirements during early developmental stages. 2–3 days old progeny with the desired genotypes were collected after eclosion and maintained at 29°C to inactivate Gal80^ts^ before dissection and immunostaining. The flies were transferred to new vials with fresh food every day. Both *UAS-dsRNA* and *UAS-shRNA* transgene stocks were used in this study. If possible, several dsRNA or shRNA lines were tested for each gene (the lines listed above showed similar phenotypes), and one or two RNAi lines were used for detailed study. Depleting *aux* in all EEs produced strong phenotype in the whole intestine, but we only focused on the R5 region for most of the analyses. The time points that the flies are analyzed/dissected were indicated in the text.

### Immunostainings and fluorescence microscopy

For standard immunostaining, intestines were dissected in 1 X PBS (10 mM NaH_2_PO_4_/Na_2_HPO_4_, 175 mM NaCl, pH 7.4) and fixed in 4% paraformaldehyde for 25 min at room temperature. Samples were rinsed, washed with 1 X PBT (0.1% Triton X-100 in 1 X PBS) and blocked in 3% BSA in 1 X PBT for 45 min. Primary antibodies were added to the samples and incubated at 4 °C overnight. The following primary antibodies were used: mouse mAb anti-Dl (C594.9B, 1:50, developed by Dr S. Artavanis-Tsakonas, Developmental Studies Hybridoma Bank (DSHB)), mouse mAb anti-Prospero (MR1A, 1:100, developed by Dr C.Q. Doe, DSHB), rabbit anti-β-glactosidase (1:5,000, Cat No: 55978, Cappel, USA), mouse anti-β-glactosidase (1:1,000, Cat No: 2372, Cell Signaling Technology, USA), rabbit anti-pH3 (pSer10, 1:2,000, Cat No: H0412, Millipore, USA), mouse anti-Slit (1:25, C555.6D, developed by Dr S. Artavanis-Tsakonas, DSHB), rabbit anti-pERK (p-p44/42, 1:200, Cat No: 4370, Cell Signaling Technology, USA), rabbit anti-GFP (1:1,000, Cat No: Ab6556, Abcam, USA), mouse anti-EGFR (clone C-273, 1:100, Cat No: E2906, Sigma-Aldrich, USA), and guinea pig anti-Aux (1:1,000) [35]. The rinsing and washing procedures were conducted and samples were then incubated with the secondary antibodies conjugated with Cy3 or 488 (1:400, Cat No: 711-165-152, 715-165-151, 711-545-152, 715-545-151, Jackson ImmunoResearch, USA) with a dilution of 1:400 for 2 h at room temperature. DAPI (Sigma-Aldrich USA, 0.1 μg/mL) was added after secondary antibody staining. The samples were mounted in mounting medium (70% glycerol containing 2.5% DABCO). Although the size of the whole midgut is increased, ISCs in the R2 and anterior midgut also over-proliferate and intestinal homeostasis is lost in the whole intestine upon Aux depletion in all EEs, only the R5 region of intestines was captured by a Zeiss LSM780 inverted confocal microscope although the intestinal homeostasis is lost in the whole *pros*^*ts*^ *> aux*^*RNAi*^ intestines, and were further processed in Adobe Photoshop and Illustrator.

### qRT-PCR

RNA was extracted from 30 brains or guts using TRIzol (Invitrogen, USA). RNA was cleaned using RNAeasy (QIAGEN, Germany), and complementary DNA (cDNA) was synthesized using the iScript cDNA synthesis kit (Bio-Rad, USA). Quantitative PCR was performed using the iScript one step RT-PCR SYBR green kit (Bio-Rad, USA). Data were acquired using an iQ5 System (Bio-Rad, USA). qRT-PCR was performed in duplicate on each of three independent biological replicates. All results are presented as mean ± SD of the biological replicates. The ribosomal gene *RpL11* was used as the normalization control.

qPCR primers:

*sli*-F: GATGCCACCACCCTTCCGGC
*sli*-R: GCATTCGCAGCTTGGTCAGCC
*RpL11*-F: GGTCCGTTCGTTCGGTATTCGC
*RpL11*-R: GGATCGTACTTGATGCCCAGATCG

### Quantification of percentage of specific EE subtypes over all EEs

To examine the percentage of specific EE subtypes over all EEs in the R5 region of intestines, different subtype-specific EE drivers such as *tkGal4*, *amonGal4*, and *386YGal4* were used. The intestines carrying such Gal4 and UAS-GFP (and or UAS-RFP) transgene were dissected and stained with mouse anti-Pros antibody (1:50, DSHB). These EE-subtype specific drivers are only expressed in some of EE subtypes, but not all EEs, along the whole intestine. The percentage of these EE subtypes over all EEs was calculated as the number of GFP+ cells/Pros+ cells. The results showed that Tk+ EEs took up to 49% of total EEs, while the percentage for amon+ EEs and 386Y+ EEs are 47% and 40%, respectively.

### Chromatin-immunoprecipitation (ChIP) and quantitative PCR (ChIP-qPCR)

About 200 *tub*^*ts*^ *> aux*^*RNAi*^*, pnt-GFP* flies (along with the same number of *tub*^*ts*^ *> luc*^*RNAi*^*, UAS-GFP* flies as control) were roughly ground on ice and 1 mL pre-chilled PBS with protease inhibitor cocktail (PBS+PIC) was added. To cross-link DNA with proteins, 37% formaldehyde was added to a final concentration of 1%. The cross-linking reaction was quenched with 0.125 M glycine-PBS for 5 min. The tissue was collected and washed with PBS+PIC for 5 min. The samples were re-suspended with 1 mL of fly lysis buffer (with PIC) and homogenized on ice to collect the nuclear pellet. The nuclear pellet was re-suspended with 1 mL RIPR lysis buffer, incubated on ice for 10 min, followed by sonication to shear genomic DNA to segments around 750 bp in length (JY 92-IIDN Focused-ultrasonicator, SCIENTZ). The fragmented chromatin was centrifuged at 13,000 rpm for 15 min and the supernatant was transferred to new tubes, 1/20 of lysate (vol/vol) was saved as input. GFP beads (Cat No: KTSM1334, KT Health, China) were added for ChIP reaction at 4°C overnight. The immunocomplexes were washed 3 times with PBT, 150 uL ChIP elution buffer was added and the samples were incubated at 65°C for 30 min. 6 uL 5M NaCl and 2 uL RNase A were added to all samples and incubated at 37°C for 30 min, followed by adding 2 uL proteinase K and de-cross-linking overnight at 65°C. The DNA was extracted by phenol/chloroform and ethanol-precipitation. The pellets were re-suspended in 20 µL TE. Quantitative PCR (qPCR) was performed using the iScript one step RT-PCR SYBR green kit (Bio-Rad, USA). Data were acquired using an iQ5 System (Bio-Rad, USA). qPCR was performed in duplicate on each of three independent biological replicates. All results are presented as mean ± SD of the biological replicates.

ChIP-qPCR primers:

*pnt*-BP1-5: CGAGTCCATAGAGATC
*pnt*-BP1-3: AAATTTCCGCCTCCCT
*pnt*-BP2-5: gtagggaaccccaaacataa
*pnt*-BP2-3: tgggcgaatttatgaagaca
*pnt*-BP3-5: TACCAATTTAATGAGCGC
*pnt*-BP 3-3: tttactgggcgactcta
*pnt*-BP4-5: GGCTTTGCAAATAGGT
*pnt*-BP4-3: ttgtgggactggcact

### Signal quantiﬁcation

ImageJ and Zen3.6 softwares were used for signal quantiﬁcation of Slit, pERK, EGFR, EGFR-GFP, and slit-lacZ fluorescence intensity. For signal quantification in cells, parameters of Area, Mean grey value, Min & Max grey value and Limit to threshold in Set Measurement of ImageJ software were selected, the Decimal places (0–9) was set at 3. At least ten different images were analyzed for each sample.

### Data analysis

The number of total cells, *pros>GFP*^+^, *esg-lacZ*^+^, *Dl-lacZ*^+^, *Gbe+Su(H)-lacZ*^+^, Dl^+^, and pH3^+^ cells were counted from confocal images with indicated genotypes using ImageJ software. Data processing was analyzed and performed using GraphPad Prism 7.0 (GraphPad Software Inc., USA). P values were determined by unpaired Student’s *t t*est (for comparisons between two groups) or Ordinary one-way ANOVA test (for comparisons among multiple groups). The graphs were further modified using Adobe Photoshop and Illustrator. All results are presented as mean ± SD. *p* > 0.05: not significant (ns), *p < 0.05, **p < 0.01, ***p < 0.001, ****p < 0.0001.

## Supporting information

S1 Fig Expression pattern of Aux in intestinal cells and double confirmation of Aux antibody specificity. (A) Aux (by Aux antibody in red) is expressed in all intestinal cell types, including EEs (by *pros>GFP* in green, white arrowheads). The *pros>GFP* and Aux channels are showed separately in black white. (B) Aux (by Aux antibody in green) is also expressed in all EEs (pan-EEs, by Pros antibody in red, white arrowheads) in intestines. The progenitors are indicated by yellow arrowheads. The region within the white box is magnified. The Pros and Aux channels are showed separately in black white. (C) The specificity of Aux antibody is further confirmed by depleting *aux* in a subtype of EEs (red, white arrowheads) using *TkGal4, UAS-RFP, tubGal80*^*ts*^ (*Tk*^*ts*^ *> RFP*). Please note that the levels of Aux protein are dramatically decreased in Tk+ EE cells. The progenitors are indicated by yellow arrowheads. The region within the white box is magnified. The RFP and Aux channels are showed separately in black white. Blue indicates DAPI staining for DNA. Scale bars: 5 μm (A), 20 μm (B and C), 2 μm (enlarged region in B and C).
(TIF)

S2 Fig *aux* depletion in EEs significantly affects life span and intestinal homeostasis. (A) The life span curves of flies with indicated genotypes. (B) The overview of the whole intestines of flies with indicated genotypes. (C) Compared to control intestines (*pros*^*ts*^ *> luc*^*RNAi*^), the number of intestinal cells (by DAPI in blue) and EEs (by *pros>GFP* in green, white arrowheads) is significantly increased in *pros*^*ts*^ *> aux*^*RNAi*^ intestines. *pros>GFP* channel is showed separately in black white. (D) Quantification of the diameter of intestines with indicated genotypes. n ≥ 10 intestines, ****p < 0.0001. (E) Quantification of the number of intestinal cells/image in intestines with indicated genotypes. Mean ± SD is shown. n ≥ 10 images, ****p < 0.0001. (F) Quantification of the number of *pros>GFP* cells/image in intestines with indicated genotypes. Mean ± SD is shown. n ≥ 10 images, ****p < 0.0001. Scale bars, 100 μm (B), 20 μm (C).
(TIF)

S3 Fig Intestines of different controls show that the defects are indeed caused by *aux* depletion in EEs. (A) Progenitors (red, by esg-lacZ) in control RNAi (*luc*^*RNAi*^) and *aux*^*RNAi*^ intestines without driver. The number of *esg-lacZ*^+^ cells are not increased compared to *pros*^*ts*^ *> aux*^*RNAi*^ intestines (please refer to Fig 1A and 1B for comparison). The esg-lacZ channel is showed separately in black white. (B) ISCs (red, by Dl) and EEs (red, by Pros) in control RNAi (*luc*^*RNAi*^) and *aux*^*RNAi*^ intestines without driver. The number of Dl^+^ and Pros^+^ cells are not increased compared to *pros*^*ts*^ *> aux*^*RNAi*^ intestines (please refer to Fig 1C-1E for comparison). The Dl and Pros channel is showed separately in black white. Scale bars: 20 μm.
(TIF)

S4 Fig Intestinal homeostasis is disrupted in *pros*^*ts*^ *> aux*^*RNAi*^ intestines. (A) Compared with control intestines (*pros*^*ts*^ *> luc*^*RNAi*^), depletion of Aux in EEs with functional RNAi constructs against *aux* leads to significant increase of progenitors (by *esg-lacZ* in red, white arrowheads). While simultaneous expression of *aux-Flag-GFP* could completely rescue the significant increase of progenitors observed in *pros*^*ts*^ *> aux*^*RNAi*^ intestines. Please refer to Fig 1B for quantification data. (B) Compared to control intestines (*pros*^*ts*^ *> luc*^*RNAi*^), the number of ISCs (by Dl in red, yellow arrowheads) and EEs (by Pros in red, white arrowheads) is significantly increased in *pros*^*ts*^ *> aux*^*RNAi*^ intestines. *pros>GFP* and Dl/Pros channels are showed separately in black white. Please refer to Fig 1C for the merged panels and Fig 1D and 1E for quantification data. (C) Compared to control intestines (*pros*^*ts*^ *> luc*^*RNAi*^), the number of ISCs (by *Dl-lacZ* in red, white arrowheads) is dramatically increased in *pros*^*ts*^ *> aux*^*RNAi*^ intestines at 29°C for 7 days. *pros>GFP* cells are in green. (D) Quantification of the number of *Dl-lacZ*^*+*^ cells/image in different genotypes indicated. Mean ± SD is shown. n ≥ 10 images, ****p < 0.0001. Please note that *pros*^*ts*^ *> aux*^*RNAi*^ intestines are highly deformed preventing accurate quantification of the number of *Dl-lacZ*^*+*^ cells. (E) Quantification of the percentage of *Dl-lacZ*^+^ cells in different genotypes indicated. Mean ± SD is shown. n ≥ 10 images, ****p < 0.0001. Scale bars: 20 μm.
(TIF)

S5 Fig Manupulation of Notch and JAK/STAT signaling in EEs by *pros*^*ts*^ driver did not result in obvious changes in intestinal homeostasis. (A) Compared to control and *aux* depletion in EEs, neither *Notch* depletion nor ectopic *hop*^*TumL*^ expression caused obvious changes in the number of *esg*+ cells (red, by esg-lacZ). Quantification of the number of *esg-lacZ*^*+*^ cells/image in different genotypes indicated. Mean ± SD is shown. n ≥ 10 images, ****p < 0.0001. *p* > 0.05, ns: not significant. (B) Compared to control and *aux* depletion in EEs, neither *Notch* depletion nor ectopic *hop*^*TumL*^ expression caused obvious changes in ISC proliferation (red, by pH3). Quantification of the number of pH3^*+*^ cells/image in different genotypes indicated. Mean ± SD is shown. n ≥ 10 images, ****p < 0.0001. *p* > 0.05, ns: not significant. Scale bars: 20 μm.
(TIF)

S6 Fig No obvious defects are observed in nSybGal80, *pros*^*ts*^ *> aux*^*RNAi*^ intestines and depleting *aux* in EEs by *CG32547Gal4* results in increased ISC proliferation. (A) As nSybGal80 is also expressed in most EEs, no obvious defects are observed in nSybGal80, *pros*^*ts*^ *> aux*^*RNAi*^ intestines. Quantification of the number of *esg-lacZ*^*+*^ cells/image and Dl^*+*^ cells/image in different genotypes indicated. Mean ± SD is shown. n ≥ 10 images, *p* > 0.05, ns: not significant. (B) Depleting *aux* in EEs by another pan-EE driver, *CG32547Gal4,* results in increased ISC proliferation (by pH3 in red). Quantification of the number of pH3^*+*^ cells/image in intestines with indicated genotypes. Mean ± SD is shown. n ≥ 10 images, ****p < 0.0001. Scale bars: 20 μm.
(TIF)

S7 Fig Neuronal Aux unlikely contributes the defects observed in *ChATGal80*, *pros*^*ts*^ *> aux*^*RNAi*^ intestines. Compared to control intestines (*ChATGal80*, *pros*^*ts*^ *> luc*^*RNAi*^), the number of progenitors (by *esg-lacZ* in red) is dramatically increased in *ChATGal80*, *pros*^*ts*^ *> aux*^*RNAi*^ intestines at 29°C for 7 days. *pros>GFP* cells are in green. Quantification of the number of *esg-lacZ*^*+*^ cells/gut surface in different genotypes indicated. Mean ± SD is shown. n ≥ 10 images, ****p < 0.0001. Scale bars: 20 μm.
(TIF)

S8 Fig Different EE subtype drivers are only expressed in a specific subtype of EEs. Co-staining of Pros (red, labelling all EEs) with different subtype EE drivers (*386YGal4 > GFP*, *amonGal4 > GFP*, and *tkGal4 > GFP*) shows that these drivers are only expressed in part of EEs. The Pros and GFP channels are showed separately in black white. Scale bars: 20 μm.
(TIF)

S9 Fig No obvious defects are observed in *tk*^*ts*^ *> aux*^*RNAi*^ intestines. (A) Compared to control intestines (*pros*^*ts*^ *> luc*^*RNAi*^), no obvious defects are observed in *tk*^*ts*^ *> aux*^*RNAi*^ intestines (by *tk > GFP* in green, white arrowheads). *tk > GFP* channel is showed separately in black white. Scale bars, 20 μm. (B) Quantification of the number of pH3+ cells/gut in different genotypes indicated. Mean ± SD is shown. n ≥ 10 intestines, ****p < 0.0001, *p* > 0.05, ns: not significant.
(TIF)

S10 Fig Slit-Robo signaling also affects ISC proliferation. (A) The number of progenitors (by esg-lacZ in red) is significantly increased in *pros*^*ts*^*>slit*^*RNAi*^. Quantification of the number of esg-lacZ+ cells/image in different genotypes indicated. Mean ± SD is shown. n ≥ 10 images, ****p < 0.0001. (B) The number of progenitors (by Dl in red) and EEs (by Pros in red) is significantly increased in *esg*^*ts*^*>robo2*^*RNAi*^ intestines. The Dl/Pros, GFP, and DAPI channels are showed separately in black white. (C) Quantification of the number of *esg > GFP*^+^ cells/image in different genotypes indicated. Mean ± SD is shown. n ≥ 10 images, ****p < 0.0001. (D) ISC proliferation is also increased in *esg*^*ts*^*>robo2*^*RNAi*^ intestines. The pH3, GFP, and DAPI channels are showed separately in black white. (E) Quantification of the number of pH3+ cells/gut in different genotypes indicated. n ≥ 10 intestines, ****p < 0.0001. The nucleus is labelled by DAPI in blue. Scale bars: 20 μm.
(TIF)

S11 Fig Aux plays minor or no roles in Slit excytosis in EEs. Compared to Slit proteins in control intestine which it can be detected in progenitors (yellow arrowhead) and EEs (white arrowhead), Slit proteins can also be detected in progenitors (yellow arrowheads) and EEs (white arrowheads) in *pros*^*ts*^ *> aux*^*RNAi*^ intestines when it is ectopically expressed. The Slit channel is showed separately in black white. Scale bars: 10μm.
(TIF)

S12 Fig EGFR-GFP is accumulated in *aux*-depleted EEs. Compared to control intestines in which EGFR (by EGFR-GFP in green) is barely detectable in EEs, the protein levels of EGFR-GFP are significantly increased in EEs of *pros*^*ts*^ *> aux*^*RNAi*^ flies (white arrowheads) at 29°C for 7 days. EGFR-GFP in progenitors/ISCs is indicated by yellow arrowheads. EGFR-GFP channel is showed separately in black white. Quantification of the fluorescence intensity of EGFR-GFP in EEs in control and *pros*^*ts*^ *> aux*^*RNAi*^ intestines. Mean ± SD is shown. n ≥ 10 cells from different intestines, ****p < 0.0001. Scale bars, 5 μm.
(TIF)

S13 Fig Tissue homeostasis is disrupted when endocytosis is blocked in EEs. (A) Compared to control intestines, when endocytosis was blocked in EEs, the number of *esg-lacZ*^+^ cells (red, white arrowheads) was significantly increased and these cells formed large clusters with varied cell sizes and many of them were polyploid, indicative of intestinal homeostasis disruption. Control and *pros*^*ts*^ *> aux*^*v22*^ panel are the same as Fig 3G respectively. Scale bars, 20 μm. (B) Compared to control intestines in which EGFR (by EGFR in green) is barely detectable in EEs (red, by RFP), EGFR proteins are significantly enriched in EEs of *pros*^*ts*^ *> Avl*^*RNAi*^ flies (white arrowheads) at 29°C for 7 days. EGFR in progenitors/ISCs is indicated by yellow arrowheads. EGFR and RFP channels are showed separately in black white. Scale bars, 5 μm.
(TIF)

S14 Fig Slit levels are regulated by EGFR signaling. (A) The protein levels of Slit proteins (red, white arrowheads indicate EEs and yellow arrowheads indicate progenitors) are diminished upon ectopic activation of EGFR signaling in EEs, while the levels are significantly increased when EGFR signaling is blocked by *Raf* knockdown. Slit channel is showed separately in black white. (B) Quantification of the fluorescence intensity of Slit in progenitors of intestines with indicated genotypes. Mean ± SD is shown. n ≥ 10 cells from different intestines, ****p < 0.0001. Scale bars: 10 μm.
(TIF)

S15 Fig The expression levels of *slit* are significantly reduced in the intestines of *pros*^*ts*^ *> aux*^*RNAi*^ flies, but largely unaffected in the brains. (A) The levels of *slit* transcripts are significantly decreased in *pros*^*ts*^ *> aux*^*RNAi*^ intestines as determined by RT-qPCR. (B) The levels of *slit* transcripts are largely unaffected in the brains of *pros*^*ts*^ *> aux*^*RNAi*^ flies as determined by RT-qPCR.
(TIF)

S16 Fig *slit* acts downstream of EGFR signaling. Ectopic activation of EGFR signaling (by Raf^gof^ expression) in EEs leads to great increase of progenitors (by *esg-lacZ* in red) at 29°C for 7 days, while co-expression of *slit* completely suppresses the increased number of progenitors. Quantification of the number of *esg-lacZ*^*+*^ cells/gut surface in different genotypes indicated. Mean ± SD is shown. n ≥ 10 images, ****p < 0.0001. Scale bars: 20 μm.
(TIF)

S17 Fig *slit* expression is de-repressed in progenitors when EGFR signaling is blocked. In control intestines, *slit* (by *slit-lacZ* in red) is expressed in EEs (small diploid *esg > GFP*^-^ cells, yellow arrowheads), while the expression of *slit* is barely detectable in progenitors (by *esg > GFP* in green, white arrowheads) in which EGFR signaling is activated. However, when EGFR signaling is blocked in progenitors (by knocking down *Raf*), the expression of *slit* (by *slit-lacZ* in red) is dramatically increased in these progenitors, indicating that the expression *slit* is suppressed by EGFR signaling. *slit-lacZ, esg > GFP*, and DAPI channels are showed separately in black white. Scale bars, 10 μm.
(TIF)

S18 Fig EGFR signaling activation in EEs (by ectopic *pntP1* expression) results in great accumulation of progenitors. Ectopic activation of EGFR signaling (by ectopic *pntP1* expression) in all EEs leads to great accumulation of progenitors (by *esg-lacZ* in red) at 29°C for 7 days. Quantification of the number of *esg-lacZ*^*+*^ cells/gut surface in different genotypes indicated. Mean ± SD is shown. n ≥ 10 images, ****p < 0.0001. Scale bars: 20 μm.
(TIF)

S19 Fig Model of Aux in EEs controlling intestinal homeostasis through cell-cell communications. In EEs of wildtype (WT) intestines, Aux associates with EGFR and constantly promotes its internalization and degradation to quench EGFR signaling, Slit-Robo signaling is activated to ensure proper ISC proliferation and progeny differentiation for intestinal homeostasis control. Whilst in intestines with *aux*-depleted EEs, EGFR can not be internalized and be accumulated on the plasma membrane of EEs, resulting in ectopic EGFR signaling. Upon ectopic activation of EGFR signaling, the transcription factor Pointed binds to the binding motifs near the TSS of *slit* and suppresses the expression of *slit*. Meanwhile, the expression of cytokine Upd3 is significantly increased in some *aux*-depleted EEs and neighboring wild type ECs, which will activate JAK/STAT signaling in ISCs for excessive ISC proliferation. Further, activated EGFR signaling also regulates multiple downstream targets in *aux-*defective EEs, including the yet-to-be identified diffusible factor(s) (termed X). The yet-to-be identified factor X from *aux*-depleted EEs may send signal to ECs, resulting death of ECs which secrete cytokine Upd3 to activate JAK/STAT signaling in ISCs to promote ISC hyper-proliferation. With the combined effects of Upd3-JAK/STAT signaling, the yet-to-be identified diffusible factor X and defective Slit-Robo signaling, ISCs, released from proliferation restraints, undergo excessive proliferation and aberrant progeny differentiation, leading to intestinal homeostasis disruption. The images within the figure panels were drew by hands.
(TIF)
