# Supplementary material for: Auxilin in enteroendocrine cells controls intestinal homeostasis through inter-cell signaling relays
Source: PLoS Genet. 2026 Oct 26;22(10):e1012353. doi: 10.1371/journal.pgen.1012353 (PMC13719025; doi:10.1371/journal.pgen.1012353)
Supplement: Supplementary file 1 [file pgen.1012353.s021.docx]

**Response to the Reviewers’ comments**

**(PGENETICS-D-26-00252-R1)**

**General response to all Reviewers**:

Dear Reviewers;

We want to thank you all for your valuable time and efforts to carefully review our previous manuscript and for the constructive comments and suggestions. We are pleased to see that all of you are supportive of our previous manuscript. Your insightful and helpful comments greatly helped us to improve the overall quality of our revised manuscript. Following your valuable comments, we updated the revised manuscript with suggested experiments and new data to further bolster our conclusions of inter-cell communications between EEs and ISCs.

One of the two key issues you concerned is how to resolve the discrepancy of defective Slit-Robo phenotypes in previous reports and its contribution to *aux* depletion phenotype here. This discrepancy can be resolved positively now. Although we found that defective Slit-Robo signaling between EEs and ISCs can result in increased ISC proliferation and aberrant progeny differentiation, the phenotype is less severe than that of *aux* depletion in EEs. Therefore, we speculated that some other effector(s) may also contribute to the excessive ISC proliferation and aberrant progeny differentiation in the absence of Aux in all EEs. Interestingly, we found that, in addition to the defective Slit-Robo pathway, the expression of Upd3 from some *aux*-depleted EEs and wild type ECs is significantly increased, which will activate JAK/STAT signaling in progenitors to promote excessive ISC proliferation. Please refer to the detailed response of this concern below.

The second central question is the specificity of the phenotype observed. We performed extra experiments to further confirm that Aux functions in all EEs to restrict excessive ISC proliferation and aberrant progeny differentiation to control intestinal homeostasis under physiological conditions. Please find the new data in our revised manuscript and details discussed in each Reviewer’s comments.

Together, we have fully addressed your comments. In the following part, we address the major and minor concerns of your comments point-by-point.

**Response to the Reviewers’ comments:**

**Comments to the Authors:
Reviewer #1:** Wang et al. identified Auxilin-EGFR-Slit/Robo as a novel cascade regulating inter-cell communication and maintaining gut homeostasis in Drosophila. The findings are overall interesting, but a few concerns need to be addressed.

We thank you for your positive and motivating evaluation of our previous manuscript. Meanwhile, we fully understand your concerns. In this revision, we added new experiments that address your concerns and bolster our main conclusions. Please ﬁnd our detailed response in the following part.

First, in Fig. 4, does pnt-GFP recapitulate EGFR signaling in the gut? What is the identity of this reporter, and what is its expression pattern? The authors need to describe it and cite the original literature. For the experiment itself, considering that less than 10% of cells are EEs in the gut, ChIP-PCR is challenging, and the authors need to provide more details in the Methods section, such as how many animals were used and what the controls were. Also, in the legends, the genotypes are not clearly delineated.

**Response:** We thank you for your critical and insightful comments. Yes, you are right that *pnt-GFP* can recapitulate EGFR signaling in the gut (please refer to Fig. R1 below), as well as in the neuronal system as reported before (Sun et al., 2020, Aging Cell, PMID: 32749068).

**Fig. R1. Pnt-GFP can recapitulate EGFR signaling in the gut.**

Left panel: control intestine, the progenitors are labelled by RFP (red). Please note that Pnt-GFP signal (white arrowhead) can be only barely detectable in progenitors, mimicking that of low EGFR signaling in progenitors under physiological conditions. RFP and Pnt-GFP channels are showed separately in black and white. Magnified region is showed on its right side. Right panel: Pnt-GFP signal (white arrowheads) is dramatically increased when Raf^gof^ is induced in progenitors (high EGFR signaling activation). Magnified region is showed on its left side for convenient comparison with control. Scale bars: 20 μm.

We are sorry that we did not clearly indicate the information of *pnt-GFP* in our previous manuscript. We are sorry for the troubles caused. *pnt-GFP* is constructed by cloning BAC clone CH321-39L02 which contains the genomic fragment including the *pnt* gene by Rebecca Spokony and Kevin White, University of Chicago. Tags including GFP and FLAG had been introduced at the C-terminal end of the *pnt* gene, therefore, *pnt-GFP* produces Pnt-GFP-Flag fusion protein under the endogenous *pnt* promoter. It can rescue *pnt^Δ88^/Df(3R)Exel9012* (Boisclair Lachance et al, 2014, Dev Biol, PMID: 24240101), indicating that the fusion protein can fully substitute endogenous Pnt protein. It is only weakly expressed in progenitors, consistent with the low EGFR signaling in progenitors, under physiological conditions (please refer to Fig. R1 above). We included these information and cited the original references in the revised manuscript (please refer to the revised manuscript).

About the ChIP-PCR, you are absolutely right that performing ChIP-PCR only in EEs is super-challenging as EEs only account for less than 10% of the total cells in the gut. We failed several times by using the gut due to trace amount of precipitated DNA. Fortunately, as we found that EGFR signaling also controls *slit* expression in other cells, in the same way as it’s in EEs (please refer to Fig. S17 in the revised manuscript), therefore, we used the whole animals to perform ChIP analysis using about 200 *tub^ts^>aux^RNAi^, pnt-GFP* flies, along with the same number of *tub^ts^>luc^RNAi^, UAS-GFP* flies as control. We are sorry that we did not clearly indicate these information in our previous manuscript, we are deeply sorry for the troubles caused. We included these information in the Methods sections in the revised manuscript (please refer to the Methods sections in the revised manuscript).

Second, it is well characterized that Slit-Robo signaling regulates cell fate of ISC differentiation but not proliferation. For instance, robo mutant clone size is comparable with wild-type by MARCM assay (Biteau, 2014). Here, the authors showed that knockdown of robo2 RNAi by esg-GAL4 increases progenitor number (Fig. S10). Is this due to differentiation failure? Also, it would also be better to verify the results using multiple independent RNAi lines to avoid potential off-target effects.

**Response:** Thank you for your constructive comments. Yes, previous publication by Biteau at al., reported the Slit-Robo signaling mainly regulates ISC progeny differentiation rather than ISC proliferation. Closer examination of previous publication indicate that the proliferation of ISCs indeed increased in the absence of Slit-Robo signaling (Nagy et al., 2017). Here we found that depleting *robo2* in progenitors indeed led to increased progenitor number. We think that the differences between previous publication and our study may be due to the different reagents used. We found that the increase of progenitors is unlikely due to differentiation failure as we found that the number of mitotic *robo2*-depleted progenitors is increased (please refer to Fig. S10 in the revised manuscript). We used three different RNAi lines against *robo2* and observed the similar defects, thus excluding the possibility of RNAi off-target effects.

Meanwhile, although we found that blocking Slit-Robo signaling between EEs to ISCs indeed results in increased number of progenitors, the phenotype is less severe compared to that of *aux* depletion in EEs (please refer to Fig. 1 and S10 in the revised manuscript). Therefore, we speculated that some other effector(s) may also contribute to the excessive ISC proliferation and aberrant progeny differentiation in the absence of Aux in all EEs. Interestingly, we found that the expression of *Upd3-lacZ*, one of the important ligand of JAK/STAT signaling, is significantly upregulated in some *aux*-depleted EEs and neighboring wild type ECs (please refer to Fig. 2B in the revised manuscript). Previous papers and our study already showed that paracrine Upd3 from dying ECs could drive excessive progenitor proliferation by activating JAK/STAT signaling in ISCs under stress conditions and *aux* depletion in ECs (PMID: 19563763, 40825946). These data indicate that inter-cell communications between EEs to ISCs (by defective Slit-Robo signaling and activated JAK/STAT signaling) and EE-EC-ISC relay (by activated JAK/STAT signaling) together control the observed excessive ISC proliferation and aberrant progeny differentiation in the absence of Aux in all EEs, illustrating the complicated inter-cell communications in controlling stem cell proliferation and tissue homeostasis. We included these new important findings in the revised manuscript, further modified the manuscript and discussed the complicate consequence of Aux loss of function in all EEs in the revised manuscript (please refer to the revised manuscript).

Third, the authors put a lot of effort into characterizing pros-GAL4, which indeed is a critical issue, since prospero itself has weak expression in progenitors. For cell-cell communication studies, clean drivers are even more critical. The authors did test another driver, CG32547-GAL4, but only in figure 1. It would be helpful if the authors could test for some of the remaining figures, at least for Figs. 4A–C.

**Response:** We thank you again for your insightful and constructive comments. Yes, we put great efforts to make sure that the phenotype observed is due to *aux* depletion in all EEs, which is critical for inter-cell communication between EEs and progenitors. We fully understand your concern. Although it is reported that Prospero is weakly expressed in progenitors, we performed co-labelling analysis of *pros>GFP* with *esg-lacZ* in wild type intestines before we started this study, we did not find co-localization of *pros>GFP* cells (EEs) with *esg-lacZ* (progenitors). Previous studies showed that depleting Notch in ISCs led to rapid ISC proliferation and progeny differentiation defect, resulting in cell clusters composed of ISCs (or ISC-like cells) and/or EEs with almost identical cell size, while overexpression of constitutively active form of Hop (Hop^TumL^) in ISCs results in rapid ISC proliferation. However, we did not observe the expected phenotype as they are manipulated by *esgGal4* in progenitors, on the contrary, the *pros^ts^>Notch^RNAi^* and *pros^ts^>hop^TumL^* intestines were largely normal as those of control flies (please refer to Fig. S5 in the revised manuscript). These results show that the observed phenotype in *pros^ts^>aux^RNAi^* intestines are indeed resulted from *aux* depletion in EEs, rather than in ISCs. Furthermore, depletion of *aux* by another pan EE driver *CG32547Gal4* showed almost identical phenotype as those of *pros^ts^>aux^RNAi^* intestines. Following your constructive comments, we further examined the levels of Slit proteins by using *CG32547Gal4* driver. The results are consistent with the phenotypes observed when *prosGal4* driver was used: the levels of Slit proteins are diminished when EGFR signaling is activated (by *aux* depletion or ectopic *Raf^gof^* expression), while the levels of Slit proteins are significantly increased when EGFR signaling is blocked (please refer to Fig. 4 and S14 in the revised manuscript). We included these new data as Fig. S14 in the revised manuscript (please refer to Fig. 4 and S14 in the revised manuscript). Together, these data show that the phenotypes observed are caused by *aux* depletion in all EEs.

Lastly, for statistical analysis, all the *n* numbers mentioned in the figure legends are not clarified. Does *n* refer to the number of cells, imaging fields, or animals?

**Response:** Thank you for pointing this out. We are sorry that we did not clearly indicate what the number (n) in the quantification analyses refers to in the Figure Legends in our previous manuscript. We are sorry for the troubles caused. We included what the number refers to in the Figure Legends in the revised manuscript (please refer to the Figure Legends in the revised manuscript).

Finally, there are a few writing errors. For example, "lifespan" is more commonly used than "life span" in the field. Meanwhile, some obvious errors were also found in Supplementary Fig. 5, i.e. “not significant, ns p > 0.05.”

**Response:** Thank you again for pointing these out. We are deeply sorry for the troubles caused. We extensively examined our revised manuscript and corrected these writing errors in the revised manuscript (please refer to the revised manuscript).

**Reviewer #2:** This manuscript identifies a potentially interesting mechanism by which Auxilin in enteroendocrine cells regulates intestinal stem cell proliferation and intestinal homeostasis through an EGFR–Slit/Robo signaling. The study provides substantial genetic and phenotypic analyses and proposes that Aux restrains EGFR signaling in enteroendocrine cells, thereby maintaining slit expression and limiting ISC proliferation. The work is conceptually interesting because it highlights non-cell-autonomous regulation of ISCs by differentiated enteroendocrine cells. However, several major issues weaken the mechanistic conclusions.

Thank you for your positive and encouraging evaluation of our previous manuscript. We fully understand your concerns. In this revision, we performed new experiments to address your concerns and bolster our main conclusions. Please ﬁnd our detailed response in the following part.

Major concerns:
1. The authors conclude that Pointed directly represses slit transcription based on ChIP-qPCR analysis. However, additional functional assays, such as reporter mutagenesis of the identified Pointed binding motifs and genetic interaction experiments, would be needed to more convincingly establish direct transcriptional repression.

**Response:** We thank you for your critical and insightful comments. We understand your concern that just by performing ChIP-qPCR analysis is not sufficient to conclude that EGFR signaling (by downstream transcription factor Pnt) represses *slit* expression in EEs. We fully agree with you on this point. Therefore, we performed more genetic analyses and genetic interactions to consolidate this conclusion. Results from our genetic analyses and genetic interactions showed that: 1) EGFR signaling is activated in the absence of Aux in EEs (please refer to Fig.3 in the revised manuscript); 2) *slit* expression and Slit proteins are diminished in *aux*-depleted EEs (please refer to Fig. 2 and 4 in the revised manuscript); 3) *slit* expression is negatively regulated by EGFR signaling (please refer to Fig. 4A-D and S14 in the revised manuscript); 4) ectopic expression of *slit* effectively rescued the defects observed in *aux*-depleted intestines and Raf^gof^ expressing intestines (please refer to Fig. 2C and S16 in the revised manuscript); 5) further blocking EGFR signaling in *aux*-depleted EEs effectively rescued the defects observed in *aux*-depleted intestines (please refer to Fig. 3G,H in the revised manuscript); 6) ectopic expression of PntP1 resembles that of aux depleted intestines (please refer to Fig. S18 in the revised manuscript). Altogether, all these data show that *slit* expression is negatively regulated by EGFR signaling. The ChIP-qPCR analysis further confirmed that *slit* expression is directly regulated by EGFR signaling. We also planned to perform reporter mutagenesis of the identified Pnt binding motifs during the course of our study, however, we found the EGFs are not expressed in S2 and S2R cells, therefore, it will be quite troublesome to transform multiple plasmids into the same S2 (or S2R) cells for luciferase analysis. Further, the transfection efficiency of S2 cells is quite low, therefore, these circumstances will prevent accurate luciferase analysis. Thus, we did not further perform *in vitro* mutagenesis analyses. While making transgenic flies is also time consuming and not cost effective, even these transgenic flies were successfully constructed, it only further proves the notion that our data already showed, ie, *slit* expression is negatively regulated by EGFR signaling. Therefore, we did not make transgenic flies carrying the mutated Pnt binding motifs to further prove this conclusion. We seek your understanding on this.

2. Since slit is proposed to function downstream of Aux and EGFR signaling, additional rescue experiments should be performed to more directly test whether slit acts downstream to rescue the observed phenotypes.

**Response:** Thank you for your constructive comments. Following your constructive comments, we performed additional rescue experiments to further examine whether *slit* acts downstream to rescue the observed phenotypes. We found that ectopic expression of *Raf^gof^* in all EEs resembles that of *aux* depletion, while co-expression of *Raf^gof^* and *slit* results in complete rescue of the phenotypes observed in *pros^ts^>Raf^gof^* intestines, further indicating that *slit* acts downstream of EGFR signaling (please refer to Fig. S16 in the revised manuscript). We included these data as Fig. S16 in the revised manuscript (please refer to Fig. S16 in the revised manuscript).

3. Most conclusions rely heavily on RNAi-mediated knockdown experiments. Additional genetic approaches, such as mutant analysis or independent validation strategies, would strengthen the conclusions.

**Response:** Thank you again for your insightful comments. As EEs are post-mitotic cells, it is impossible to perform classical mosaic analyses using mutants in EEs like in ISCs. Further, it’s also not possible to examine the role of Aux in all EEs using MARCM clone technique or G-Tracing analysis, ie, analyzing the function of *aux* (or other genes) from the differentiated cells (ie EEs) from mutant clones as *aux* defective ISCs are hyper-proliferative, preventing the analysis of inter-cell communication between EEs and ISCs. Although the recently developed Flip Flop technique from Hugo Bellen lab can perform mosaic analysis in post-mitotic cells, however, it still need particular lines and mutants which are not available for *aux*. Nevertheless, we utilized different RNAi lines and performed multiple genetic analysis and genetic interactions to further confirm that Aux functions in all EEs to control ISC proliferation and progeny differentiation through synergetic effects of activated JAK/STAT signaling and defective Slit-Robo signaling (please refer to the revised manuscript).

4. In Fig. 1, expression of aux-RNAi in enteroendocrine cells induces ISC overproliferation (approximately 4 PH3+ cells per image). Because there are substantial regional differences along the intestine, it would be more appropriate to quantify the total number of PH3+ cells per intestine rather than per image.

**Response:** Thank you so much for your critical comments. We fully understand your concern. Although there are substantial regional differences along the intestine under normal conditions, we found that depleting Aux in all EEs results in homeostasis disruption due to dramatic increase of progenitor proliferation in all the regions of the intestine (please refer to Fig. S2B and others in the revised manuscript). Further, our study focused on the R5 region of the posterior midgut for phenotypic analyses and subsequent characterizations, the quantification in R5 region over the quantification of whole intestine is part vs whole, which will draw similar conclusions in the end. Therefore, our quantification in the R5 region can exclude region differences and the regional differences do not interfere with the accuracy of our conclusions. Furthermore, we also quantified the number of pH3 per intestine for some quantifications in the revise manuscript (please refer to Fig. S9B in the revised manuscript). The conclusion from the whole intestine quantification is the same as the conclusion from the R5 region quantification, further supporting our notion that focusing on the R5 region only can exclude regional differences (please refer to Fig. S9B and 1I-J in the revised manuscript).

Minor concerns:
1. The manuscript would benefit from a clearer schematic model summarizing the proposed Aux–EGFR–Slit/Robo signaling pathway and its effects on ISC proliferation and intestinal homeostasis.

**Response:** Thank you again for your constructive comment. Our conclusion is further supported by the new findings, thus we modified our schematic model accordingly. In wildtype intestines, Aux in EEs associates with EGFR and constantly promotes its internalization and degradation to quench EGFR signaling, Slit-Robo signaling is activated to ensure proper ISC proliferation and progeny differentiation for intestinal homeostasis control. Whilst in intestines with *aux*-depleted EEs, EGFR can not be internalized and be accumulated on the plasma membrane of EEs, resulting in ectopic EGFR signaling. Upon ectopic activation of EGFR signaling, the transcription factor Pointed binds to the binding motifs near the TSS of *slit* and suppresses the expression of *slit*. Meanwhile, the expression of cytokine Upd3 is significantly increased in some *aux*-depleted EEs and neighboring wild type ECs, which will activate JAK/STAT signaling in ISCs for excessive ISC proliferation. Activated EGFR signaling also regulates multiple downstream targets in *aux*-defective EEs, including the yet-to-be identified diffusible factor(s) (termed X). The yet-to-be identified factor X from *aux*-depleted EEs may send signal to ECs, resulting death of ECs which then secrete cytokine Upd3 to activate JAK/STAT signaling in ISCs to promote ISC hyper-proliferation. With the combined effects of Upd3-JAK/STAT signaling, the yet-to-be identified diffusible factor X and defective Slit-Robo signaling, ISCs are released from proliferation restraint and undergo excessive proliferation and aberrant progeny differentiation, leading to intestinal homeostasis disruption (please refer to the modified Graphic Abstract and the revised manuscript).

2. Several conclusions are overstated relative to the presented data, particularly regarding the proposed “double suppressive cascade.” For example, the authors conclude that Aux restrains ISC proliferation through an Aux–EGFR–Slit/Robo signaling cascade originating from enteroendocrine cells. However, the current data primarily show correlations among aux depletion, ectopic EGFR activation, reduced slit expression, and ISC overproliferation, rather than definitive causal relationships.

**Response:** Thank you again for your insightful comments. Yes, you are absolutely right that some conclusions are overstated relative to the presented data in our previous manuscript, we are sorry for this. As responded above, with the inclusion of new findings, we extensively modified our conclusions and schematic model to avoid overstatement in the revised manuscript (please refer to the responses above and the revised manuscript).

**Reviewer #3:** In this manuscript, the authors present an unknown function of Auxilin (Aux), a DnaJ-domain ATPase Hsc70 chaperone that regulates clathrin dynamics, in adult Drosophila midgut secretory cells. Specifically, they provide evidence that Aux is expressed in all midgut enteroendocrine cells (EEs) and non-autonomously restricts intestinal stem cell (ISC) proliferation. EE-specific aux silencing leads to ISC proliferation, accumulation of aberrantly differentiated progenitors and dysplasia. The authors go ahead to show that Aux functions in EEs to control Slit production, which is subsequently secreted and reaches its receptor Robo-2 in the ISCs to control mitosis. Interestingly, EE-specific aux silencing leads to accumulation of EGFR in the EEs (EGFR is absent from EEs under physiological conditions) which regulates slit expression through its effector transcription factor Pointed (Pnt) that binds on the enhancer of slit. This interesting paper explores an unknown role of Aux in secretory midgut cells and presents a model of EE-ISC communication through mitosis regulation through slit abundance control by EGFR signaling. Nevertheless, some issues need to be resolved before publication:

We thank you for your supportive and encouraging evaluation of our previous manuscript. In this revision, we performed new experiments to address your concerns and support our main conclusions. Please ﬁnd our detailed response in the following part.

1. In addition to the midgut EEs (and other midgut cell types), Aux is also expressed and functions in the brain. A major issue with the paper is the claim that the effects observed in the midgut via pros-Gal4 are caused only due to manipulation in the midgut EEs. To exclude the possibility that the effects observed using this driver are caused by brain cells, the authors should use pros-Gal4ts Chat-Gal80, that was shown to suppress pros-Gal4 brain expression (Medina et al., Dev. Cell 2025) with no effects on midgut EEs, to at least repeat some key experiments of aux silencing.

**Response:** Thank you for your critical and constructive comments. Yes, you are absolutely right that Aux is almost ubiquitously expressed and *prosGal4* is also expressed on the brain. We fully understand your concern that the observed phenotypes in *pros^ts^>aux^RNAi^* intestines may be remotely contributed by depletion of Aux in the brain. Although we found that the expression levels of *slit* were unaffected in the brains of *pros^ts^>aux^RNAi^* animals, further excluding the possibility that the defects observed in *pros^ts^>aux^RNAi^* intestines are due to *aux* depletion in the CNS (please refer to Fig. S15 in the revised manuscript). Following your constructive suggestion, we further depleted *aux* by *prosGal4*, in the presence of *ChATGal80* to block *prosGal4* expression in the brain, and examined the phenotype of *aux*-depleted intestines. The *ChATGal80, pros^ts^>aux^RNAi^* intestines mimic to that of *pros^ts^>aux^RNAi^* intestines (please refer to Fig. S7 in the revised manuscript). These data further support our conclusion that the defects observed in *pros^ts^>aux^RNAi^* intestines are due to *aux* depletion in all EEs, rather than its depletion in the brain and other cell types. We included these data as Fig. S7 in the revised manuscript (please refer to Fig. S7 in the revised manuscript).

2. Following up on comment #1, the authors use CG32547-Gal4, a new pan-EE driver to support their pros-Gal4 experimental results. In the original paper describing the line, there is no information about assessment of CG32547-Gal4 expression in other tissues. Have the authors tested whether CG32547-Gal4 is only expressed in the gut EEs?

**Response:** Thank you so much for your constructive comments. Yes, you are right that there is no information about the assessment of *CG32547-Gal4* expression pattern in other tissues. We roughly examined the expression pattern of *CG32547-Gal4* driver and found that it is relatively specifically expressed: it is expressed in all EEs in the gut, as well as in the brain and follicles in the ovary (please refer to Fig. R2 below). We indicated this information in the Methods section of the revised manuscript (please refer to the Methods section of the revised manuscript).

**Fig.R2. *CG32547Gal4* is also expressed in the brain and follicles of the ovary.**

As responded above, we fully understand your concern and as further blocking *prosGal4* expression in the brain did not affect the phenotype observed in *pros^ts^>aux^RNAi^* intestines, thus excluding the possibility of Aux depletion in the brain contributes to the defects observed in *pros^ts^>aux^RNAi^* intestines. Consistent with the notion that depletion of Aux in all EEs affects Slit protein levels, we found that depleting *aux* in all EEs by *CG32547-Gal4* driver also diminished the levels of Slit (please refer to Fig. S14 in the revised manuscript). Together, these data show that Aux functions in EE to control intestinal homeostasis under physiological conditions. Therefore, we did not further block the expression of *CG32547-Gal4* driver in the brain and examined the phenotype of *CG32547^ts^>aux^RNAi^* intestine again.

3. The authors claim that aux needs to be depleted by all EEs to exhibit its effects. They test different Gal4 drivers expressed in EE subsets (~40-50% of total EEs according to their quantifications) and state that no phenotype similar to pros-Gal4>auxRNAi is observed. In which part of the midgut are the EE-subset drivers expressed? On page 10, the authors state that they tested driver expression only in the posterior R5 region. Maybe the lack of phenotype is because of regional differences in Aux function and/or regional expression of the EE-subset driver. Is Aux required throughout the midgut? Or is the phenotype restricted to the R5 region?

**Response:** Thank you again for your insightful comments. Yes, it is true that only when *aux* is depleted in all EEs, can we observed the strong defects. For the expression pattern of the other EE-subtype drivers used, they are all expressed along the whole intestine, the only difference with *prosGal4* driver is that they only expression in some EEs along the whole intestine, ie, these EE-subtype specific drivers are only expressed in some EE subtypes, but not all EEs, at any region of the gut examined. We are sorry that we did not clearly indicate this in our previous manuscript and sorry for the trouble caused. We indicated this in the revised manuscript (please refer to the Methods section in the revised manuscript).

It should be mentioned that depleting *aux* in all EEs produced strong defects in the whole intestine, but not restricted to the R5 region. The reason we compared the percentage of EE subtypes in the R5 region is that we only focused on the R5 region of the posterior midgut for most of the analyses (except length of the intestine and life span), instead of randomly picking a region for confocal imaging and analysis. To avoid misunderstandings, we indicated this in the Methods section of the revised manuscript (please refer to the Methods section of the revised manuscript). The lack of obvious defects by other EE-subtype specific drivers is not because of regional differences as there is no phenotype along the whole intestine upon *aux* depletion using these EE-subtype specific drivers.

4. Along the above lines and comment #3, on page 10 and Fig. S7, the authors state that they tested 3 EE-subset drivers for expression and used 2 of them to silence aux, but on Fig. S8, they only show data for tk-Gal4. The images shown here only indicate EEs in the midgut in control and tk-Gal4>auxRNAi midguts, but it is not clear what is quantified. The most sensitive assay to detect dysplasia in the gut is overall mitosis. The authors should measure pH3-positive cells in the whole midguts of the aux-silenced flies using the different EE-specific Gal4 lines and compare the results to pan-EE Gal4 silencing.

**Response:** Thank you again for your constructive comments. There several ways to determine the phenotype upon gene silencing in EEs, you are right that counting cells undergoing mitosis is the most sensitive way. Following your constructive comments, we further quantified the number of pH3+ cells in the whole intestine of the *aux*-depleted flies using different EE-specific Gal4 lines, including *prosGal4,* *tkGal4* and *386YGal4*. The results are consistent with the notion that depleting *aux* in all EEs (by *prosGal4*) results in intestinal homeostasis disruption due to dramatically increased ISC proliferation, while depleting *aux* in specific EE subtypes results in no obvious defects (by *tkGal4* or *386YGal4*) (please refer to Fig. S9B in the revised manuscript). We included these new data as Fig. S9B in the revised manuscript (please refer to Fig. S9B in the revised manuscript).

5. EGFR is present in EEs when aux is silenced. The authors suggest that Aux promotes EGFR internalization and degradation. In Fig. S12, they show that EE-specific silencing of the endocytic gene Avl causes dysplasia. Does Avl loss impinge on EGFR localization in EEs? It would be nice to show EGFR protein in Avl-silenced EEs.

**Response:** Thank you again for your insightful comments. Yes, you are right that Avl loss impinge on EGFR localization in EEs. The results showed that, compared to control EEs in which EGFR proteins are barely detectable, EGFR proteins are enriched in *Avl*-depleted EEs, similar to those of *aux*-depleted EEs (please refer to Fig. S13B in the revised manuscript). We included these new data as Fig. S13B in the revised manuscript (please refer to pages 13-14 and Fig. S13B in the revised manuscript).

6. In all graphs measuring mitotic cells, measurements should be done for the whole midguts, unless Aux has a regional effect, which should be explicitly stated in the manuscript.

**Response:** Thank you so much for your critical comments. We fully understand your concern. Although there are substantial regional differences along the intestine, we found that depleting Aux in all EEs results in homeostasis disruption due to dramatic increase of progenitor proliferation in all the regions of the intestine (please refer to Fig. S2B and others in the revised manuscript). Further, our study focused on the R5 region of the posterior midgut for phenotypic analyses and subsequent characterizations, the quantification in R5 region over the quantification of whole intestine is part vs whole, which will draw similar conclusions in the end, therefore, our quantification in the R5 region will exclude region differences and the regional differences do not interfere with the accuracy of our conclusions. Furthermore, we also quantified the number of pH3 per intestine for some quantifications in the revise manuscript (please refer to Fig. S9B in the revised manuscript). The conclusion from the whole intestine quantification is the same as the conclusion from the R5 region, further support our notion that only focusing on the R5 region can exclude regional differences.

7. In all graphs quantifying certain cell populations (e.g., Fig. 1B, D, G, E, J and others throughout the manuscript), the cells of interest should be counted as “number of cells/gut surface”. Quantifying “number of cells/image” is misleading because the gut surface varies significantly in the images.

**Response:** Thank you again for your constructive comments. Following your constructive comments, we changed the labelling to “number of cells/gut surface” in all the related Figures to avoid misleading in the revised manuscript (please refer to the revised manuscript).

8. The text will benefit from editing for English. For example, in the abstract it is ok to say “Pan-EE aux depletion”, but it is not ok to say “in aux-depleted pan-EEs”; in the latter case, it could be phrased as “when aux is depleted from all EEs”.

**Response:** Thank you so much for pointing these out. We extensively modified the revised manuscript and revised the phrases (please refer to the revised manuscript).
