# Supplementary material for: Auxilin in enteroendocrine cells controls intestinal homeostasis through inter-cell signaling relays
Source: PLoS Genet. 2026 Oct 26;22(10):e1012353. doi: 10.1371/journal.pgen.1012353 (PMC13719025; doi:10.1371/journal.pgen.1012353)
Supplement: Peer review history

# Peer review: Decision Letter 0

**DOI:** 10.1371/journal.pgen.1012353.r001

**Authors:** Giovanni Bosco, Jason Karpac

22 May 2026

PGENETICS-D-26-00252

Auxilin in enteroendocrine cells controls intestinal homeostasis through inter-cell signaling relay

PLOS Genetics

Dear Dr. Li,

Thank you for submitting your manuscript to PLOS Genetics. After careful consideration, we feel that it has merit but does not fully meet PLOS Genetics's publication criteria as it currently stands. Therefore, we invite you to submit a revised version of the manuscript that addresses the points raised during the review process.

Please submit your revised manuscript by Jul 21 2026 11:59PM. If you will need more time than this to complete your revisions, please reply to this message or contact the journal office at plosgenetics@plos.org. When you're ready to submit your revision, log on to https://www.editorialmanager.com/pgenetics/ and select the 'Submissions Needing Revision' folder to locate your manuscript file.

Please include the following items when submitting your revised manuscript:

* A letter that responds to each point raised by the editor and reviewer(s). You should upload this letter as a separate file labeled 'Response to Reviewers'. This file does not need to include responses to any formatting updates and technical items listed in the 'Journal Requirements' section below.

* A marked-up copy of your manuscript that highlights changes made to the original version. You should upload this as a separate file labeled 'Revised Manuscript with Track Changes'.

* An unmarked version of your revised paper without tracked changes. You should upload this as a separate file labeled 'Manuscript'.

If you would like to make changes to your financial disclosure, competing interests statement, or data availability statement, please make these updates within the submission form at the time of resubmission. Guidelines for resubmitting your figure files are available below the reviewer comments at the end of this letter.

As the corresponding author, your ORCID iD is verified in the submission system and will appear in the published article. PLOS supports the use of ORCID, and we encourage all coauthors to register for an ORCID iD and use it as well. Please encourage your coauthors to verify their ORCID iD within the submission system before final acceptance, as unverified ORCID iDs will not appear in the published article. Only the individual author can complete the verification step; PLOS staff cannot verify ORCID iDs on behalf of authors.

We look forward to receiving your revised manuscript.

Kind regards,

Jason Karpac

Academic Editor

PLOS Genetics

Giovanni Bosco

Section Editor

PLOS Genetics

Aimée Dudley

Editor-in-Chief

PLOS Genetics

Anne Goriely

Editor-in-Chief

PLOS Genetics

**Journal Requirements:**

1) We have noticed that you have uploaded Supporting Information files, but you have not included a list of legends. Please add a full list of legends for your Supporting Information files after the references list.

2) Please amend your detailed Financial Disclosure statement. This is published with the article. It must therefore be completed in full sentences and contain the exact wording you wish to be published.

- State the initials, alongside each funding source, of each author to receive each grant. For example: "This work was supported by the National Institutes of Health (####### to AM; ###### to CJ) and the National Science Foundation (###### to AM)."

- State what role the funders took in the study. If the funders had no role in your study, please state: "The funders had no role in study design, data collection and analysis, decision to publish, or preparation of the manuscript.".

If you did not receive any funding for this study, please simply state: u201cThe authors received no specific funding for this work.u201d

**Reviewers' comments:**

Reviewer's Responses to Questions

**Comments to the Authors:**

**Please note here if the review is uploaded as an attachment.**

Reviewer #1: Wang et al. identified Auxilin-EGFR-Slit/Robo as a novel cascade regulating inter-cell communication and maintaining gut homeostasis in Drosophila. The findings are overall interesting, but a few concerns need to be addressed.

First, in Fig. 4, does pnt-GFP recapitulate EGFR signaling in the gut? What is the identity of this reporter, and what is its expression pattern? The authors need to describe it and cite the original literature. For the experiment itself, considering that less than 10% of cells are EEs in the gut, ChIP-PCR is challenging, and the authors need to provide more details in the Methods section, such as how many animals were used and what the controls were. Also, in the legends, the genotypes are not clearly delineated.

Second, it is well characterized that Slit-Robo signaling regulates cell fate of ISC differentiation but not proliferation. For instance, robo mutant clone size is comparable with wild-type by MARCM assay (Biteau, 2014). Here, the authors showed that knockdown of robo2 RNAi by esg-GAL4 increases progenitor number (Fig. S10). Is this due to differentiation failure? Also, it would also be better to verify the results using multiple independent RNAi lines to avoid potential off-target effects.

Third, the authors put a lot of effort into characterizing pros-GAL4, which indeed is a critical issue, since prospero itself has weak expression in progenitors. For cell-cell communication studies, clean drivers are even more critical. The authors did test another driver, CG32547-GAL4, but only in figure 1. It would be helpful if the authors could test for some of the remaining figures, at least for Figs. 4A–C.

Lastly, for statistical analysis, all the *n* numbers mentioned in the figure legends are not clarified. Does *n* refer to the number of cells, imaging fields, or animals?

Finally, there are a few writing errors. For example, "lifespan" is more commonly used than "life span" in the field. Meanwhile, some obvious errors were also found in Supplementary Fig. 5, i.e. “not significant, ns p > 0.05.”

Reviewer #2: This manuscript identifies a potentially interesting mechanism by which Auxilin in enteroendocrine cells regulates intestinal stem cell proliferation and intestinal homeostasis through an EGFR–Slit/Robo signaling. The study provides substantial genetic and phenotypic analyses and proposes that Aux restrains EGFR signaling in enteroendocrine cells, thereby maintaining slit expression and limiting ISC proliferation. The work is conceptually interesting because it highlights non-cell-autonomous regulation of ISCs by differentiated enteroendocrine cells. However, several major issues weaken the mechanistic conclusions.

Major concerns:

1. The authors conclude that Pointed directly represses slit transcription based on ChIP-qPCR analysis. However, additional functional assays, such as reporter mutagenesis of the identified Pointed binding motifs and genetic interaction experiments, would be needed to more convincingly establish direct transcriptional repression.

2. Since slit is proposed to function downstream of Aux and EGFR signaling, additional rescue experiments should be performed to more directly test whether slit acts downstream to rescue the observed phenotypes.

3. Most conclusions rely heavily on RNAi-mediated knockdown experiments. Additional genetic approaches, such as mutant analysis or independent validation strategies, would strengthen the conclusions.

4. In Fig. 1, expression of aux-RNAi in enteroendocrine cells induces ISC overproliferation (approximately 4 PH3+ cells per image). Because there are substantial regional differences along the intestine, it would be more appropriate to quantify the total number of PH3+ cells per intestine rather than per image.

Minor concerns:

1. The manuscript would benefit from a clearer schematic model summarizing the proposed Aux–EGFR–Slit/Robo signaling pathway and its effects on ISC proliferation and intestinal homeostasis.

2. Several conclusions are overstated relative to the presented data, particularly regarding the proposed “double suppressive cascade.” For example, the authors conclude that Aux restrains ISC proliferation through an Aux–EGFR–Slit/Robo signaling cascade originating from enteroendocrine cells. However, the current data primarily show correlations among aux depletion, ectopic EGFR activation, reduced slit expression, and ISC overproliferation, rather than definitive causal relationships.

Reviewer #3: In this manuscript, the authors present an unknown function of Auxilin (Aux), a DnaJ-domain ATPase Hsc70 chaperone that regulates clathrin dynamics, in adult Drosophila midgut secretory cells. Specifically, they provide evidence that Aux is expressed in all midgut enteroendocrine cells (EEs) and non-autonomously restricts intestinal stem cell (ISC) proliferation. EE-specific aux silencing leads to ISC proliferation, accumulation of aberrantly differentiated progenitors and dysplasia. The authors go ahead to show that Aux functions in EEs to control Slit production, which is subsequently secreted and reaches its receptor Robo-2 in the ISCs to control mitosis. Interestingly, EE-specific aux silencing leads to accumulation of EGFR in the EEs (EGFR is absent from EEs under physiological conditions) which regulates slit expression through its effector transcription factor Pointed (Pnt) that binds on the enhancer of slit. This interesting paper explores an unknown role of Aux in secretory midgut cells and presents a model of EE-ISC communication through mitosis regulation through slit abundance control by EGFR signaling. Nevertheless, some issues need to be resolved before publication:

1. In addition to the midgut EEs (and other midgut cell types), Aux is also expressed and functions in the brain. A major issue with the paper is the claim that the effects observed in the midgut via pros-Gal4 are caused only due to manipulation in the midgut EEs. To exclude the possibility that the effects observed using this driver are caused by brain cells, the authors should use pros-Gal4ts Chat-Gal80, that was shown to suppress pros-Gal4 brain expression (Medina et al., Dev. Cell 2025) with no effects on midgut EEs, to at least repeat some key experiments of aux silencing.

2. Following up on comment #1, the authors use CG32547-Gal4, a new pan-EE driver to support their pros-Gal4 experimental results. In the original paper describing the line, there is no information about assessment of CG32547-Gal4 expression in other tissues. Have the authors tested whether CG32547-Gal4 is only expressed in the gut EEs?

3. The authors claim that aux needs to be depleted by all EEs to exhibit its effects. They test different Gal4 drivers expressed in EE subsets (~40-50% of total EEs according to their quantifications) and state that no phenotype similar to pros-Gal4>auxRNAi is observed. In which part of the midgut are the EE-subset drivers expressed? On page 10, the authors state that they tested driver expression only in the posterior R5 region. Maybe the lack of phenotype is because of regional differences in Aux function and/or regional expression of the EE-subset driver. Is Aux required throughout the midgut? Or is the phenotype restricted to the R5 region?

4. Along the above lines and comment #3, on page 10 and Fig. S7, the authors state that they tested 3 EE-subset drivers for expression and used 2 of them to silence aux, but on Fig. S8, they only show data for tk-Gal4. The images shown here only indicate EEs in the midgut in control and tk-Gal4>auxRNAi midguts, but it is not clear what is quantified. The most sensitive assay to detect dysplasia in the gut is overall mitosis. The authors should measure pH3-positive cells in the whole midguts of the aux-silenced flies using the different EE-specific Gal4 lines and compare the results to pan-EE Gal4 silencing.

5. EGFR is present in EEs when aux is silenced. The authors suggest that Aux promotes EGFR internalization and degradation. In Fig. S12, they show that EE-specific silencing of the endocytic gene Avl causes dysplasia. Does Avl loss impinge on EGFR localization in EEs? It would be nice to show EGFR protein in Avl-silenced EEs.

6. In all graphs measuring mitotic cells, measurements should be done for the whole midguts, unless Aux has a regional effect, which should be explicitly stated in the manuscript.

7. In all graphs quantifying certain cell populations (e.g., Fig. 1B, D, G, E, J and others throughout the manuscript), the cells of interest should be counted as “number of cells/gut surface”. Quantifying “number of cells/image” is misleading because the gut surface varies significantly in the images.

8. The text will benefit from editing for English. For example, in the abstract it is ok to say “Pan-EE aux depletion”, but it is not ok to say “in aux-depleted pan-EEs”; in the latter case, it could be phrased as “when aux is depleted from all EEs”.

**********

**Have all data underlying the figures and results presented in the manuscript been provided?**

Large-scale datasets should be made available via a public repository as described in the *PLOS Genetics*  data availability policy, and numerical data that underlies graphs or summary statistics should be provided in spreadsheet form as supporting information.

Reviewer #1: Yes

Reviewer #2: Yes

Reviewer #3: Yes

**********

PLOS authors have the option to publish the peer review history of their article (what does this mean?). If published, this will include your full peer review and any attached files.

If you choose “no”, your identity will remain anonymous but your review may still be made public.

**Do you want your identity to be public for this peer review?**  For information about this choice, including consent withdrawal, please see our Privacy Policy.

Reviewer #1: No

Reviewer #2: No

Reviewer #3: No

[NOTE: If reviewer comments were submitted as an attachment file, they will be attached to this email and accessible via the submission site. Please log into your account, locate the manuscript record, and check for the action link "View Attachments". If this link does not appear, there are no attachment files.]

**Figure resubmission:**

While revising your submission, we strongly recommend that you use PLOS’s NAAS tool (https://ngplosjournals.pagemajik.ai/artanalysis) to test your figure files. NAAS can convert your figure files to the TIFF file type and meet basic requirements (such as print size, resolution), or provide you with a report on issues that do not meet our requirements and that NAAS cannot fix.

After uploading your figures to PLOS’s NAAS tool - https://ngplosjournals.pagemajik.ai/artanalysis, NAAS will process the files provided and display the results in the "Uploaded Files" section of the page as the processing is complete. If the uploaded figures meet our requirements (or NAAS is able to fix the files to meet our requirements), the figure will be marked as "fixed" above. If NAAS is unable to fix the files, a red "failed" label will appear above. When NAAS has confirmed that the figure files meet our requirements, please download the file via the download option, and include these NAAS processed figure files when submitting your revised manuscript.

**Reproducibility:**

To enhance the reproducibility of your results, we recommend that authors of applicable studies deposit laboratory protocols in protocols.io, where a protocol can be assigned its own identifier (DOI) such that it can be cited independently in the future. Additionally, PLOS ONE offers an option to publish peer-reviewed clinical study protocols. Read more information on sharing protocols at https://plos.org/protocols?utm_medium=editorial-email&utm_source=authorletters&utm_campaign=protocols

# Author comment: Author response to Decision Letter 1

**DOI:** 10.1371/journal.pgen.1012353.r002

18 Sep 2026

Attachment Submitted filename: Response to the Reviewers comments.docx

# Peer review: Decision Letter 1

**DOI:** 10.1371/journal.pgen.1012353.r003

**Authors:** Giovanni Bosco, Jason Karpac

14 Oct 2026

Dear Dr Li,

We are pleased to inform you that your manuscript entitled "Auxilin in enteroendocrine cells controls intestinal homeostasis through inter-cell signaling relays" has been editorially accepted for publication in PLOS Genetics. Congratulations!

Before your submission can be formally accepted and sent to production you will need to complete our formatting changes, which you will receive in a follow up email. Please be aware that it may take several days for you to receive this email; during this time no action is required by you. Please note: the accept date on your published article will reflect the date of this provisional acceptance, but your manuscript will not be scheduled for publication until the required changes have been made.

Once your paper is formally accepted, an uncorrected proof of your manuscript will be published online ahead of the final version, unless you’ve already opted out via the online submission form. If, for any reason, you do not want an earlier version of your manuscript published online or are unsure if you have already indicated as such, please let the journal staff know immediately at plosgenetics@plos.org.

In the meantime, please log into Editorial Manager at https://www.editorialmanager.com/pgenetics/, click the "Update My Information" link at the top of the page, and update your user information to ensure an efficient production and billing process. Note that PLOS requires an ORCID iD for all corresponding authors. Therefore, please ensure that you have an ORCID iD and that it is validated in Editorial Manager. To do this, go to ‘Update my Information’ (in the upper left-hand corner of the main menu), and click on the Fetch/Validate link next to the ORCID field.  This will take you to the ORCID site and allow you to create a new iD or authenticate a pre-existing iD in Editorial Manager.

If you have a press-related query, or would like to know about making your underlying data available (as you will be aware, this is required for publication), please see the end of this email. If your institution or institutions have a press office, please notify them about your upcoming article at this point, to enable them to help maximise its impact. Inform journal staff as soon as possible if you are preparing a press release for your article and need a publication date.

Thank you again for supporting open-access publishing; we are looking forward to publishing your work in PLOS Genetics!

Yours sincerely,

Jason Karpac

Academic Editor

PLOS Genetics

Giovanni Bosco

Section Editor

PLOS Genetics

Aimée Dudley

Editor-in-Chief

PLOS Genetics

Anne Goriely

Editor-in-Chief

PLOS Genetics

www.plosgenetics.org

BlueSky: @plos.bsky.social

----------------------------------------------------

Comments from the reviewers (if applicable):

Reviewer's Responses to Questions

**Comments to the Authors:**

**Please note here if the review is uploaded as an attachment.**

Reviewer #2: The authors have addressed most of my previous concerns and substantially strengthened the manuscript. One point still requires clarification. The revised manuscript states that Pnt directly binds the slit regulatory region and suppresses slit transcription. While the ChIP-qPCR results support Pnt binding and the genetic data support negative regulation of slit by EGFR/Pnt signaling, the current data do not directly demonstrate that Pnt binding itself mediates transcriptional repression. I suggest that the authors moderate this conclusion accordingly.

Reviewer #3: The authors have responded to all my comments and provided a sufficiently revised manuscript.

**********

**Have all data underlying the figures and results presented in the manuscript been provided?**

Large-scale datasets should be made available via a public repository as described in the *PLOS Genetics*  data availability policy, and numerical data that underlies graphs or summary statistics should be provided in spreadsheet form as supporting information.

Reviewer #2: Yes

Reviewer #3: Yes

**********

PLOS authors have the option to publish the peer review history of their article (what does this mean?). If published, this will include your full peer review and any attached files.

If you choose “no”, your identity will remain anonymous but your review may still be made public.

**Do you want your identity to be public for this peer review?**  For information about this choice, including consent withdrawal, please see our Privacy Policy.

Reviewer #2: **Yes:**  Aiguo Tian

Reviewer #3: **Yes:**  Chrysoula Pitsouli

----------------------------------------------------

**Data Deposition**

If you have submitted a Research Article or Front Matter that has associated data that are not suitable for deposition in a subject-specific public repository (such as GenBank or ArrayExpress), one way to make that data available is to deposit it in the Dryad Digital Repository. As you may recall, we ask all authors to agree to make data available; this is one way to achieve that. A full list of recommended repositories can be found on our website.

The following link will take you to the Dryad record for your article, so you won't have to re‐enter its bibliographic information, and can upload your files directly:

http://datadryad.org/submit?journalID=pgenetics&manu=PGENETICS-D-26-00252R1

More information about depositing data in Dryad is available at http://www.datadryad.org/depositing. If you experience any difficulties in submitting your data, please contact help@datadryad.org for support.

Additionally, please be aware that our data availability policy requires that all numerical data underlying display items are included with the submission, and you will need to provide this before we can formally accept your manuscript, if not already present.

----------------------------------------------------

**Press Queries**

If you or your institution will be preparing press materials for this manuscript, or if you need to know your paper's publication date for media purposes, please inform the journal staff as soon as possible so that your submission can be scheduled accordingly. Your manuscript will remain under a strict press embargo until the publication date and time. This means an early version of your manuscript will not be published ahead of your final version. PLOS Genetics may also choose to issue a press release for your article. If there's anything the journal should know or you'd like more information, please get in touch via plosgenetics@plos.org.

# Editor report: Acceptance letter

**DOI:** 10.1371/journal.pgen.1012353.r004

**Authors:** Giovanni Bosco, Jason Karpac

PGENETICS-D-26-00252R1

Auxilin in enteroendocrine cells controls intestinal homeostasis through inter-cell signaling relays

Dear Dr Li,

We are pleased to inform you that your manuscript entitled "Auxilin in enteroendocrine cells controls intestinal homeostasis through inter-cell signaling relays" has been formally accepted for publication in PLOS Genetics! Your manuscript is now with our production department and you will be notified of the publication date in due course.

The corresponding author will soon be receiving a typeset proof for review, to ensure errors have not been introduced during production. Please review the PDF proof of your manuscript carefully, as this is the last chance to correct any errors. Please note that major changes, or those which affect the scientific understanding of the work, will likely cause delays to the publication date of your manuscript.

Soon after your final files are uploaded, unless you have opted out or your manuscript is a front-matter piece, the early version of your manuscript will be published online. The date of the early version will be your article's publication date. The final article will be published to the same URL, and all versions of the paper will be accessible to readers.

For Research Articles, you will receive an invoice from PLOS for your publication fee after your manuscript has reached the completed accept phase. If you receive an email requesting payment before acceptance or for any other service, this may be a phishing scheme. Learn how to identify phishing emails and protect your accounts at https://explore.plos.org/phishing.

Thank you again for supporting PLOS Genetics and open-access publishing. We are looking forward to publishing your work!

With kind regards,

Janani Seenivasan

PLOS Genetics

On behalf of:

The PLOS Genetics Team

Carlyle House, Carlyle Road, Cambridge CB4 3DN | United Kingdom

plosgenetics@plos.org | +44 (0) 1223-442823

plosgenetics.org | Twitter: @PLOSGenetics
